# RNA-binding protein FXR1 is presented in rat brain in amyloid form

**DOI:** 10.1038/s41598-019-55528-6

**Published:** 2019-12-12

**Authors:** Julia V. Sopova, Elena I. Koshel, Tatiana A. Belashova, Sergey P. Zadorsky, Alexandra V. Sergeeva, Vera A. Siniukova, Alexandr A. Shenfeld, Maria E. Velizhanina, Kirill V. Volkov, Anton A. Nizhnikov, Daniel V. Kachkin, Elena R. Gaginskaya, Alexey P. Galkin

**Affiliations:** 1 St. Petersburg Branch, Vavilov Institute of General Genetics, St. Petersburg, Russian Federation; 2 Department of Genetics and Biotechnology, St. Petersburg State University, St. Petersburg, Russian Federation; 3 Laboratory of Amyloid Biology, St. Petersburg State University, St. Petersburg, Russian Federation; 4 Department of Cytology and Histology, St. Petersburg State University, St. Petersburg, Russian Federation; 5 SCAMT laboratory, ITMO University, St. Petersburg, Russian Federation; 6 Laboratory of signal regulation, All-Russia Research Institute for Agricultural Microbiology, Pushkin, St. Petersburg, Russian Federation; 7 Research Resource Center “Molecular and Cell Technologies”, St. Petersburg, Russian Federation; 8 Laboratory for Proteomics of Supra-Organismal Systems, All-Russia Research Institute for Agricultural Microbiology, Pushkin, St. Petersburg, Russia

**Keywords:** Protein aggregation, Molecular neuroscience

## Abstract

Amyloids are β-sheets-rich protein fibrils that cause neurodegenerative and other incurable human diseases affecting millions of people worldwide. However, a number of proteins is functional in the amyloid state in various organisms from bacteria to humans. Using an original proteomic approach, we identified a set of proteins forming amyloid-like aggregates in the brain of young healthy rats. One of them is the FXR1 protein, which is known to regulate memory and emotions. We showed that FXR1 clearly colocalizes in cortical neurons with amyloid-specific dyes Congo-Red, Thioflavines S and T. FXR1 extracted from brain by immunoprecipitation shows yellow-green birefringence after staining with Congo red. This protein forms in brain detergent-resistant amyloid oligomers and insoluble aggregates. RNA molecules that are colocalized with FXR1 in cortical neurons are insensitive to treatment with RNase A. All these data suggest that FXR1 functions in rat brain in amyloid form. The N-terminal amyloid-forming fragment of FXR1 is highly conserved across mammals. We assume that the FXR1 protein may be presented in amyloid form in brain of different species of mammals, including humans.

## Introduction

Amyloids are non-branching fibrils that are composed of stacked monomers stabilized by intermolecular β-sheets. Such extra- and intracellular fibrils formed by various proteins were discovered in bacteria and eukaryotes^1,2^. Some proteins form pathological amyloid fibrils that cause neurodegenerative and other incurable human diseases, called amyloidoses, that affect millions of people worldwide^2^. Other amyloids regulate vital processes. Functional amyloids in bacteria contribute to biofilm development and can generate toxic oligomers that cause damage to lipid membranes^3^. A repeat domain of human Pmel17 protein forms fibrils essential for melanin deposition and synthesis in human pigment-specific cells^4^. It was shown that mammalian neuropeptides and protein hormones are stored in an amyloid-like cross β-sheet-rich conformation in the pituitary secretory granules^5^. There is reason to assume that functional amyloids can play a role in the regulation of memory. For example, proteins of the CPEB family form oligomers critical for the persistence of long-term synaptic facilitation and long-term memory in the brains of *Aplysia californica, Drosophila melanogaster* and *Mus musculus*. These oligomers, like classical amyloids, are resistant to treatment with sodium dodecyl sulfate (SDS)^6–8^. However, SDS-resistance is a typical feature not only of amyloids, but also of some other filaments and protein complexes of a non-amyloid nature^9,10^. Besides, oligomers of CPEB bind amyloid-specific dyes only *in vitro* or when overexpressed *in vivo* but not under native conditions^6^. Thus, the amyloid nature of the CPEB proteins under native conditions remains controversial.

Discovery of each new functional amyloid is a notable scientific event because until recently there were no methods for large-scale screening for amyloids. Recent advances in the development of a methodology of proteomic screening for amyloids allow to move from identifying individual amyloid proteins to systemic analysis of the prevalence and significance of amyloids in different species^9–12^. These methods are based on the resistance of amyloid aggregates to treatment with SDS that makes it possible to separate them from most other non-amyloid protein complexes^13^. The amyloid properties of the proteins identified in such screenings should be confirmed by further individual analysis.

Here, we applied our original proteomic approach in order to search for functional amyloid-forming proteins in the brains of young healthy rats. We identified several proteins that formed amyloid-like aggregates in brain and performed in-depth analysis of the amyloid properties of RNA-binding protein FXR1, which is involved in the regulation of memory and emotions^14,15^. This protein contains RNA-binding motives (KH1/KH2 and RGG) and differentially regulates RNA translation and stability^16,17^. Small FXR1-conaining RNP granules facilitate translation in growth-arrest conditions, but in dividing cell culture FXR1 forms insoluble aggregates that cause translation silencing^18,19^. We demonstrated that FXR1 forms both, amyloid oligomers and insoluble aggregates in rat cortical neurons. Amylod conformers of FXR1 in brain cortex colocalized with mRNA molecules that are resistant to RNase treatment. Our data suggest that amyloid structures play a role in the regulation of physiological processes in the mammalian brain.

## Results

### Proteomic screening identifies proteins forming detergent-resistant amyloid-like aggregates in the brain of *Rattus norvegicus*

To identify the proteins forming detergent-resistant amyloid-like aggregates in the brains of six-month-old males of *Rattus norvegicus*, protein lysate was obtained from the total brain samples. The fraction of proteins forming SDS-resistant aggregates was isolated and purified using the PSIA-LC-MALDI proteomic approach^9^ optimized for brain tissues. The proteins present in this fraction were solubilized, treated with trypsin, separated by HPLC and identified by mass spectrometry. The experiment was repeated independently with brain samples obtained from three animals. The proteins NSF, MBP, RIMS1, STXB1 and FXR1 were identified by mass-spectrometry in all three analyzed samples (Table 1; Supplementary Figs. S1–S5). All of these proteins, except MBP, contain potentially amyloidogenic regions predicted by the ArchСandy algorithm^20^ (Table 1). Notably, there are some data suggesting that the myelin basic protein (MBP), that is a component of specialized membrane covering axons, may form amyloid structures in the brain^21^.

**Table 1:** The proteins forming SDS-resistant aggregates in rat brain.

Protein	Function	Amyloidogenic regions of the protein as predicted by ArchCandy
NSF	Catalyzes the fusion of transport vesicles within the Golgi cisternae and is required for transport from the endoplasmic reticulum to the Golgi stack^41^)	118–145, 168–186, 350–374
MBP	Has a role in the formation and stabilization of myelin layers^42^	—
RIMS1	Involved in exocytosis, may act as scaffold protein that regulates neurotransmitter release at the active zone^43^	758–780, 1538–1569
STXB1	Participates in the regulation of synaptic vesicle docking and fusion and is essential for neurotransmission^44^	264–276, 405–430, 537–573
FXR1	RNA-binding protein^17^, involved in regulation of long-term memory, mood and emotion^14,15^	2–20, 225–247

In this paper we focused on the analysis of the amyloid properties of the FXR1 protein that belongs to the Fragile X-Related (FXR) family of RNA-binding proteins, which also includes Fragile X Mental Retardation (FMRP) and Fragile X-Related 2 (FXR2) proteins^22^. The FXR2 and FMRP proteins, in contrast to FXR1, were not identified in SDS-resistant fractions. The FXR1 protein identified in our screen binds specific RNAs and is involved in regulation of long-term memory and emotion processing^14,15^. Taking into consideration these neurospecific functions of FXR1, we decided to comprehensively analyze its amyloid properties *in vivo* and *in vitro*.

### FXR1 forms amyloid conformers in brain and colocalizes with mRNA molecules that are resistant to RNAse treatment

**Figure 1:**
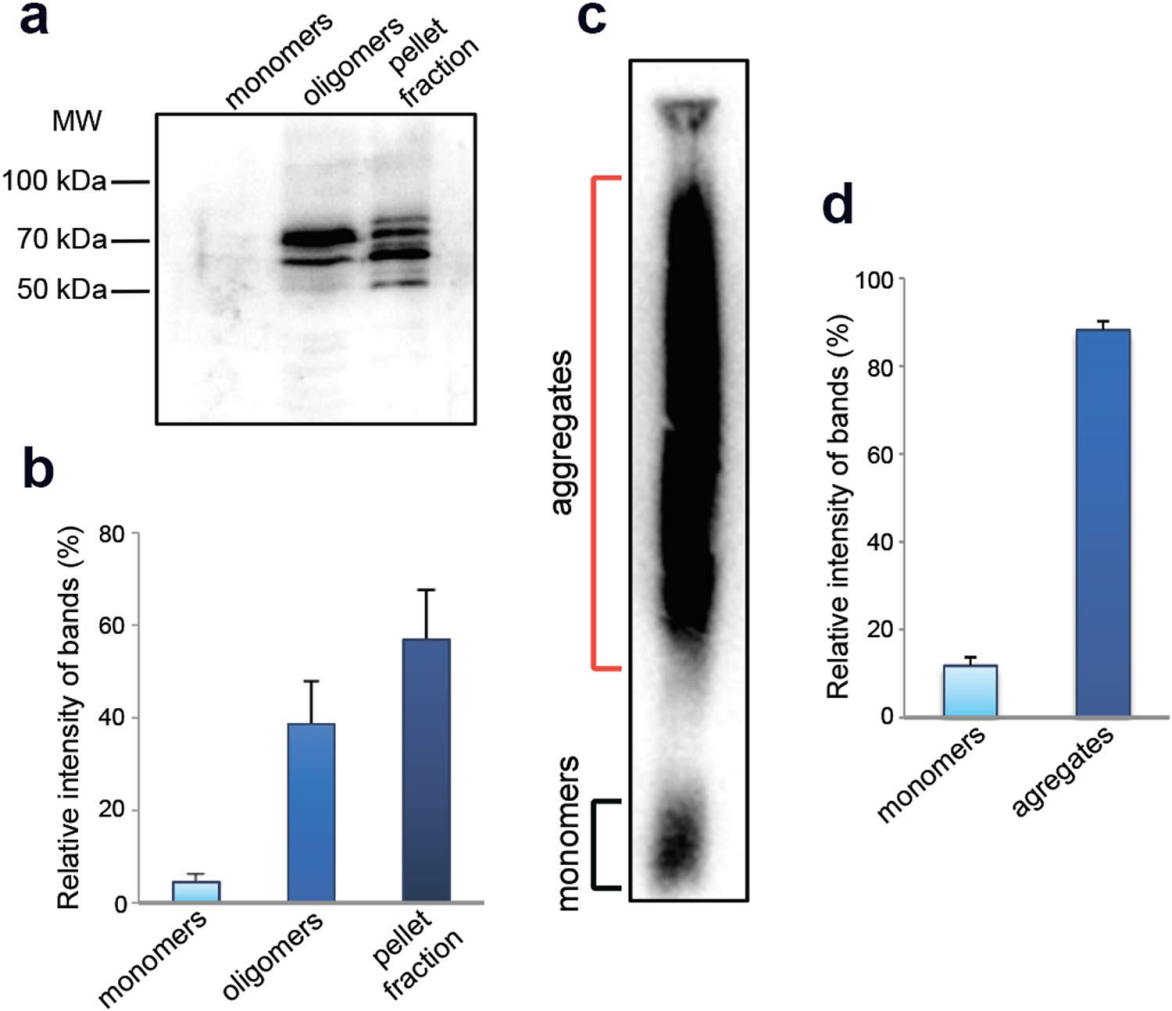
FXR1 forms SDS-resistant oligomers and aggregates in rat brain. (**a**) FXR1 is present in brain in fraction of oligomers and insoluble aggregates. Total protein lysate was divided into fractions of monomers less than 100 kDa, oligomers larger than 100 kDa, and insoluble aggregates. The fractions were subjected to SDD-PAGE and analyzed by immunoblotting with anti-FXR1 antibodies. (**b**) Relative intensity of bands corresponding to FXR1 monomers, oligomers and insoluble aggregates is represented as mean ± SEM for three independent brain samples. (**c**) A large portion of FXR1 in rat brain forms SDS-resistant aggregates. Total rat brain lysate was treated with1% SDS at RT, subjected to SDD-AGE, and analyzed by immunoblotting with anti-FXR1 antibodies. (**d**) Relative intensity of bands corresponding to FXR1 monomers and SDS-resistant aggregates is represented as mean ± SEM for three independent brain samples.

To check whether FXR1 aggregates *in vivo*, total protein lysate from the rat brain was separated into three fractions: (1) proteins less than 100 kDa; (2) soluble proteins larger than 100 kDa; (3) insoluble aggregates. Using an FXR1-specific antibody we demonstrated that this protein is predominantly present in fractions containing oligomers and insoluble aggregates (Fig. 1a,b). Monomers of FXR1 were almost undetectable in the brain lysate (Fig. 1a). Two bands are detected in the oligomeric fraction and four bands are found in the fraction containing insoluble aggregates (Fig. 1a). These results are in complete agreement with previously published data, according to which several isoforms of FXR1 protein exist in the mouse and rat brains due to alternative splicing of 3′ part of FXR1 mRNA^23–25^.

Known amyloids form SDS-resistant aggregates *in vivo* that may be detected by semi-denaturing detergent agarose gel electrophoresis (SDD-AGE)^26,27^. The total brain lysate was treated with 1% SDS and separated by agarose gel electrophoresis. A large portion of FXR1 formed detergent-insoluble aggregates (Fig. 1c,d). This result resembles the data of proteomic screening for amyloid-forming proteins according to that FXR1 forms SDS-resistant aggregates *in vivo* in all rat brain samples analyzed (Supplementary Fig. S5).

**Figure 2:**
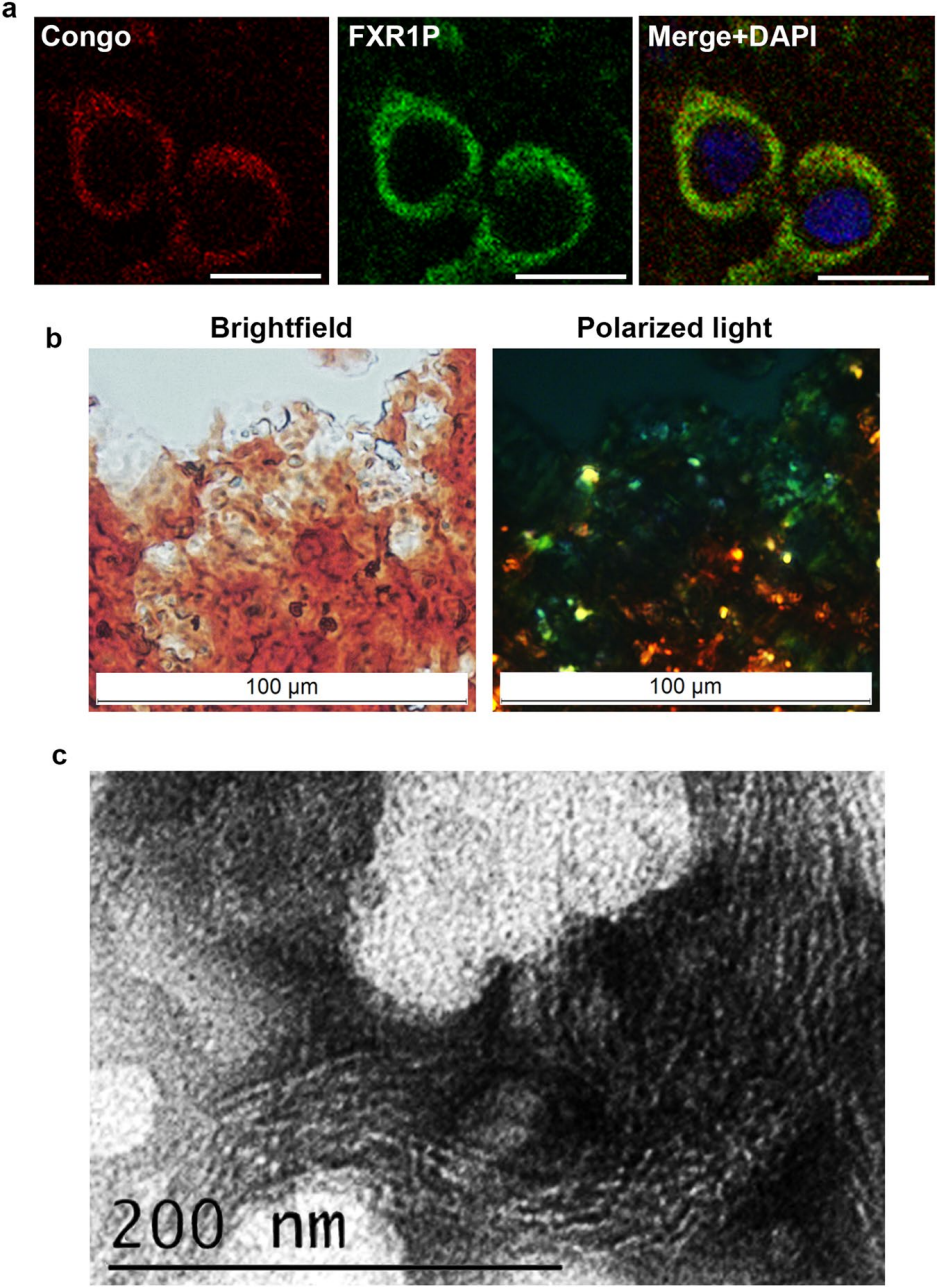
FXR1 colocalizes with amyloid-specific dye Congo red in cortical neurons and demonstrates amyloid properties. (**a**) FXR1 is present in the perinuclear cytoplasm of cortical neurons and colocalizes with amyloid-specific dye Congo red. Scale bar, 10 µm. See also Supplementary Fig. S6. (**b**) FXR1 extracted from rat brain using immunoprecipitation binds Congo red and demonstrates yellow-green birefringence under polarized light. Scale bar, 100 μM (**c**) Fibrils of FXR1 extracted from rat brain using immunoprecipitation visualized by TEM. Scale bar, 200 nm.

To verify that FXR1 is present in amyloid form in brain, we compared the localization of FXR1 with the localization of amyloid-specific dyes Congo Red, Thioflavin S and Thioflavin T on cryosections of the brain cortex of young rats. The endogenous FXR1 protein was detected in the perinuclear cytoplasm of cortical neurons (Fig. 2a). The amyloid-specific dye Congo red was detected by confocal microscopy as described previously^28^. The location of FXR1 precisely coincided with the signals of Congo red. Colocalization of FXR1 and Congo red was estimated using Pearson’s coefficient for 100 random cells, shown as mean ± SEM (Supplementary Fig. S6). Pearson correlation coefficient was 0,72 ± 0,05. However, we did not detect the yellow-green birefringence seen under crossed polarized light. The yellow-green birefringence produced by Congo red stained deposits is a gold standard for amyloids detection. This approach is applicable to detect extracellular deposits or large condensed intracellular amyloid granules. We suggested that the sensitivity of this method is not sufficient for detection of the FXR1 conformers in the cytoplasm of neurons. To test this hypothesis, FXR1 was extracted from brain by immunoprecipitation, concentrated by centrifugation and immediately stained with Congo Red. The results presented in the Fig. 2b shows that FXR1 immunoprecipitated from brain binds Congo red and demonstrates yellow-green birefringence under crossed polarized light. Fibrils of FXR1 extracted from rat brain using immunoprecipitation were visualized by TEM (Fig. 2c).

**Figure 3:**
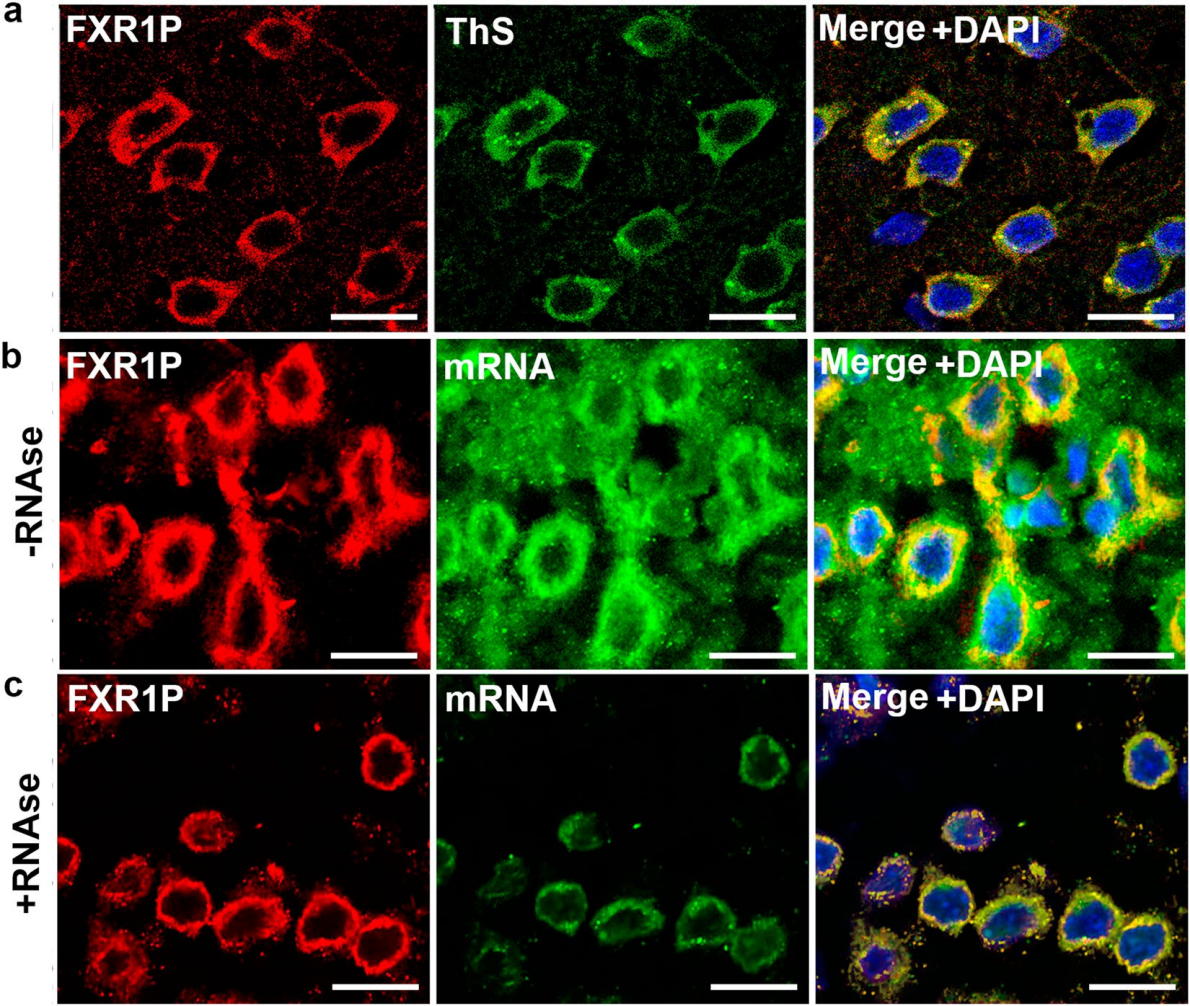
FXR1 colocalizes with amyloid-specific dye Thioflavin S in cortical neurons and colocalizes with mRNA molecules resistant to RNAse treatment. (**a**) FXR1 is present in the perinuclear cytoplasm of cortical neurons and colocalizes with Thioflavin S. See also Supplementary Fig. S6. (**b**) FXR1 colocalizes with some portion of mRNA in the cytoplasm of cortical neurons. (**c**) mRNAs that are colocalized with FXR1 are detected after treatment with RNAse A, whereas other mRNAs degrade. Immunohistochemistry and fluorescent *in situ* hybridization on the cryosections of the rat brain cortex were carried out as described in “Materials and methods”. Scale bar for sections (**a**–**c**) is 20 µm.

FXR1 also colocalizes in cortical neurons with amyloid-specific dyes Thioflavin S (Figs. 3a and S6) and Thioflavin T (Supplementary Fig. S7). Taking into consideration that FXR1 forms SDS-resistant aggregates, colocalizes with all three amyloid specific dyes and shows yellow-green birefringence under crossed polarized light, we can conclude that this protein is present in rat brain in amyloid form under native conditions.

FXR1 is known to bind different RNA molecules and affects their stability and translation efficiency^17^. To analyze the binding of FXR1 with mRNA, the brain cryosections were hybridized with biotinylated poly-dT, subsequently visualized by avidin-Alexa Fluor 488 and immunostained with anti-FXR1 antibody. As expected, we demonstrated that FXR1 colocalizes with a portion of mRNA in the cytoplasm of cortical neurons, whereas an essential part of mRNA is not associated with this protein (Fig. 3b). To check stability of mRNA molecules colocalized with amyloid conformers of FXR1, cryosections of the brain cortex were treated with RNase A in extremely high concentration (500 μg/ml). Then cryosections were hybridized with poly-dT and immunostained with anti FXR1-antibody. Molecules of mRNA that are colocalized with FXR1 were clearly detected after treatment with RNase A, whereas other mRNA molecules had completely degraded (Fig. 3c).

### Aggregation of the FXR1 protein depends on its N-terminal region

**Figure 4:**
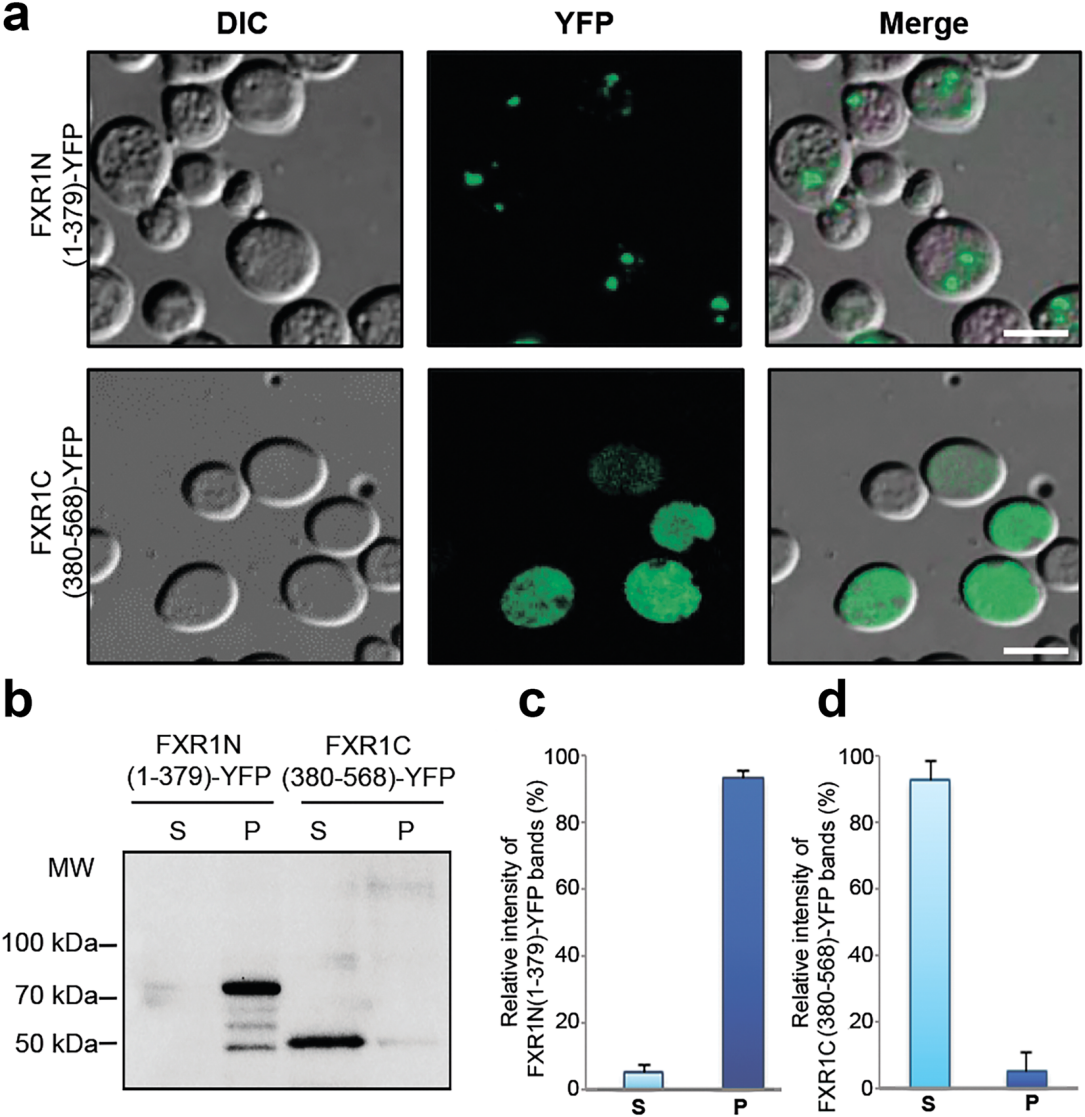
Amyloid aggregation of FXR1 protein depends on its N-terminal region. (**a**) The FXR1N(1-379)-YFP protein forms visible aggregates in yeast cells, whereas FXR1C(380-568)-YFP is evenly distributed in cytoplasm. Scale bar, 10 µm. Three independently obtained transformants expressing the FXR1N(1-379)-YFP and FXR1C(380-568)-YFP proteins were included in the analysis. About one hundred cells of each transformant were analyzed. (**b**) Protein lysates expressing the FXR1N(1-379)-YFP and FXR1C(380-568)-YFP proteins were centrifuged, separated into the soluble and insoluble fractions and analyzed by immunoblotting. The FXR1N(1-379)-YFP protein forms insoluble aggregates, whereas the FXR1C(380-568)-YFP is present in soluble form. P – pellet fraction; S - supernatant fraction. (**c**,**d**) - Relative intensities of bands corresponding to monomers and aggregates of FXR1N(1-379)-YFP and FXR1C(380-568)-YFP represented as mean ± SEM, for three independent protein samples.

Data obtained using the ArchСandy algorithm^20^ show that the N-terminal part of FXR1 contains potentially amyloidogenic regions (Table 1). However, bioinformatic data are only predictive and require experimental verification. To identify the regions of FXR1 responsible for its aggregation, the sequences encoding FXR1(1-379 aa) and FXR1(380–568 aa) were PCR amplified from the total cDNA obtained from the brain-extracted mRNA of Wistar rats and fused in frame with the sequence encoding yellow fluorescent protein (YFP). The chimeric genes *FXR1N(1-379)-YFP* and *FXR1C(380-568)-YFP* were expressed under the control of the *CUP1* promoter in the *Saccharomyces cerevisiae* strain BY4742. As expected, FXR1N(1-379)-YFP formed dot-like fluorescent foci in yeast cells (Fig. 4a). The FXR1C(380-568)-YFP protein exhibited diffuse fluorescence in the yeast cytoplasm (Fig. 4a). Protein lysates were obtained from yeast cells expressing the chimeric proteins, centrifuged and separated into the soluble and insoluble fractions. The FXR1N(1-379)-YFP protein was predominantly present in insoluble fraction, whereas the FXR1C(380-568)-YFP was detected in soluble fraction (Fig. 4b–d). Thus, the differential centrifugation analysis coupled with Western blot confirms the fluorescent microscopy data.

**Figure 5:**
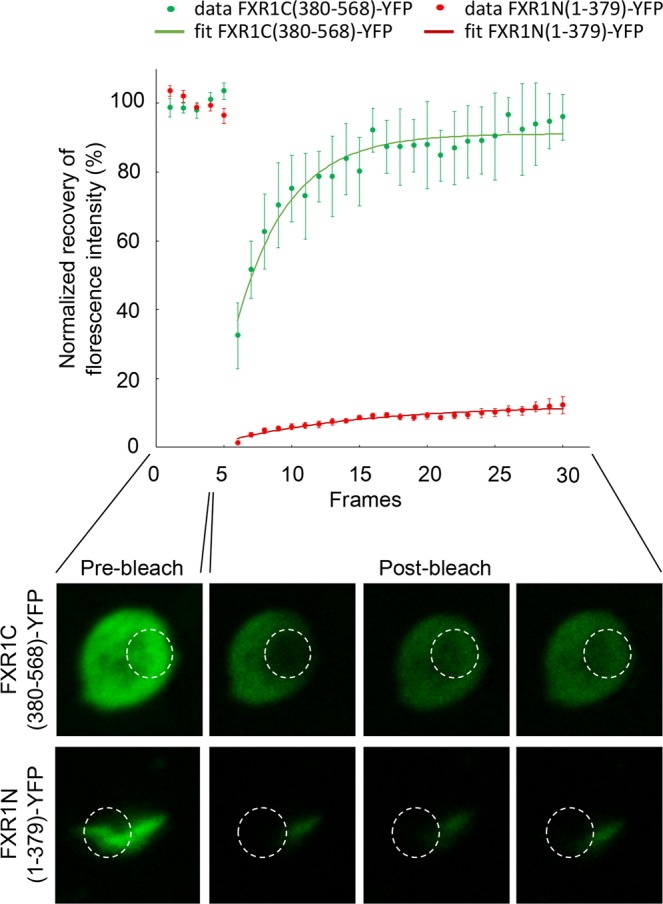
FRAP microscopy of the chimeric FXR1N(1-379)-YFP and FXR1C(380-568)-YFP proteins in yeast cells. On the graph, normalized fluorescence recoveries (%) for the corresponding proteins and their fits according to a simple monoexponential model are presented. Data are shown as the means ± SEM (sample set *n* = 5). Images below the graph demonstrate ROIs (white circles) in yeast cells expressing studied proteins before (pre-bleach) and after photobleaching (1st, 15th and 25th post-bleach frames, 25 post-bleach frames in total, time between each frame = 30 sec).

In order to check whether the FXR1(1-379)-YFP chimeric protein, like other amyloids, forms solid, immobile aggregates, the FRAP analysis was performed. The FXR1(1-379)-YFP protein located inside the fluorescent aggregates, unlike FXR1(380-568)-YFP, is virtually immobile (Fig. 5). This is characteristic of dense, compact structures, in particular, amyloids. A slight restoration of fluorescence intensity is usually explained by the dynamics of small oligomers, which have some mobility.

**Figure 6:**
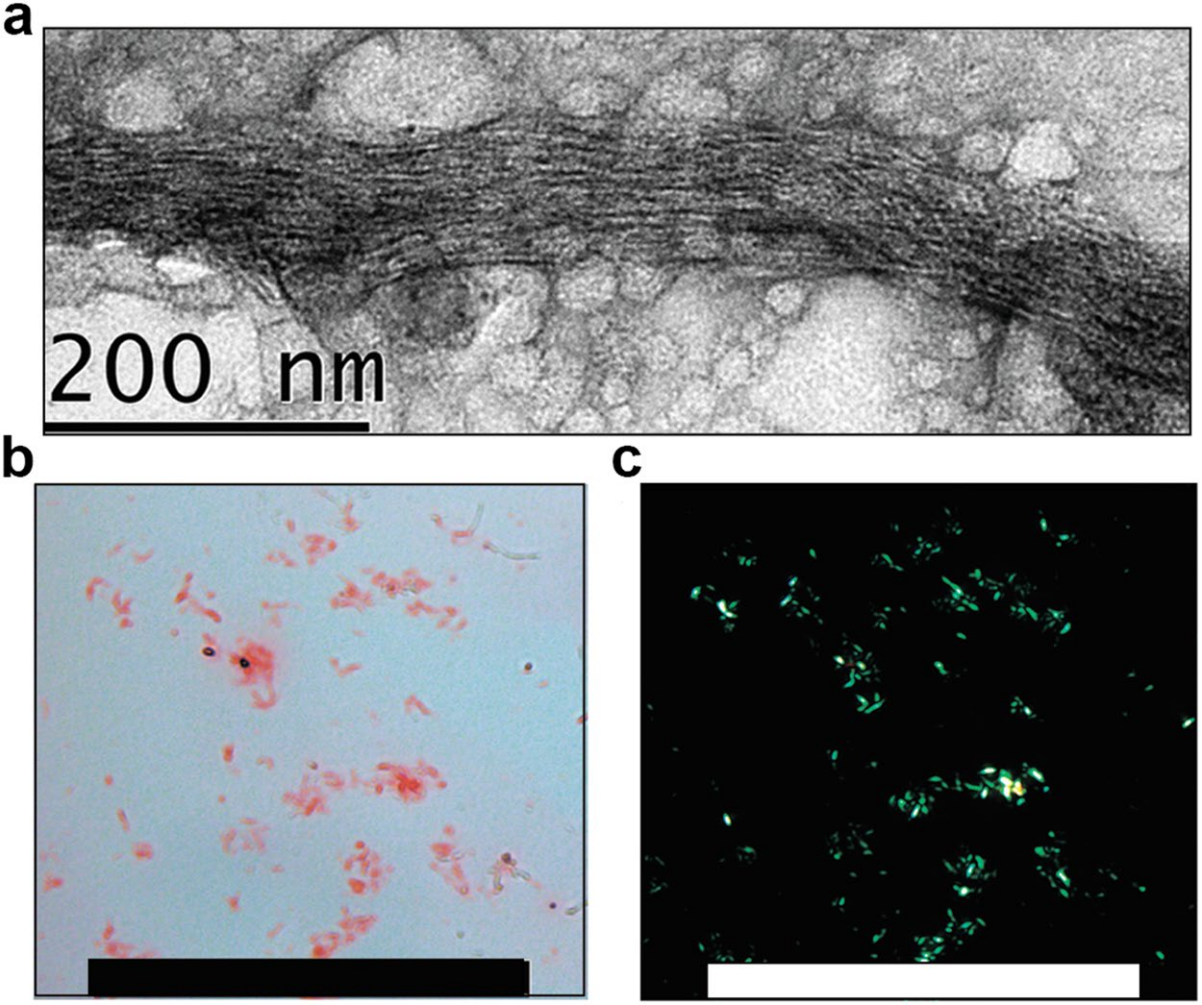
Recombinant protein 6His-FXR1(1-379) demonstrates amyloid properties *in vitro*. (**a**) The 6xHis-FXR1(1-379) recombinant protein was incubated for 48 hours at 37 °C in Tris buffer at 37 °C with slow rotation and analyzed by TEM. Scale bar, 200 nm. (**b**) Fibrils of 6xHis-FXR1(1-379) bind CR and (**c**) display yellow-green birefringence when viewed between crossed polarizers. Scale bar, 100 μM.

The amyloid properties of the N-terminal fragment of FXR1 were also analyzed *in vitro*. The 6xHis-FXR1(1-379) recombinant protein immediately aggregated when placed into Tris buffer at 37 °C, however microscopically discernible fibrils were detected only after 48 hours of incubation with slow rotation (Fig. 6a). These fibrils bound Congo Red (Fig. 6b) and manifested yellow-green birefringence when assayed by polarization microscopy (Fig. 6c).

**Figure 7:**
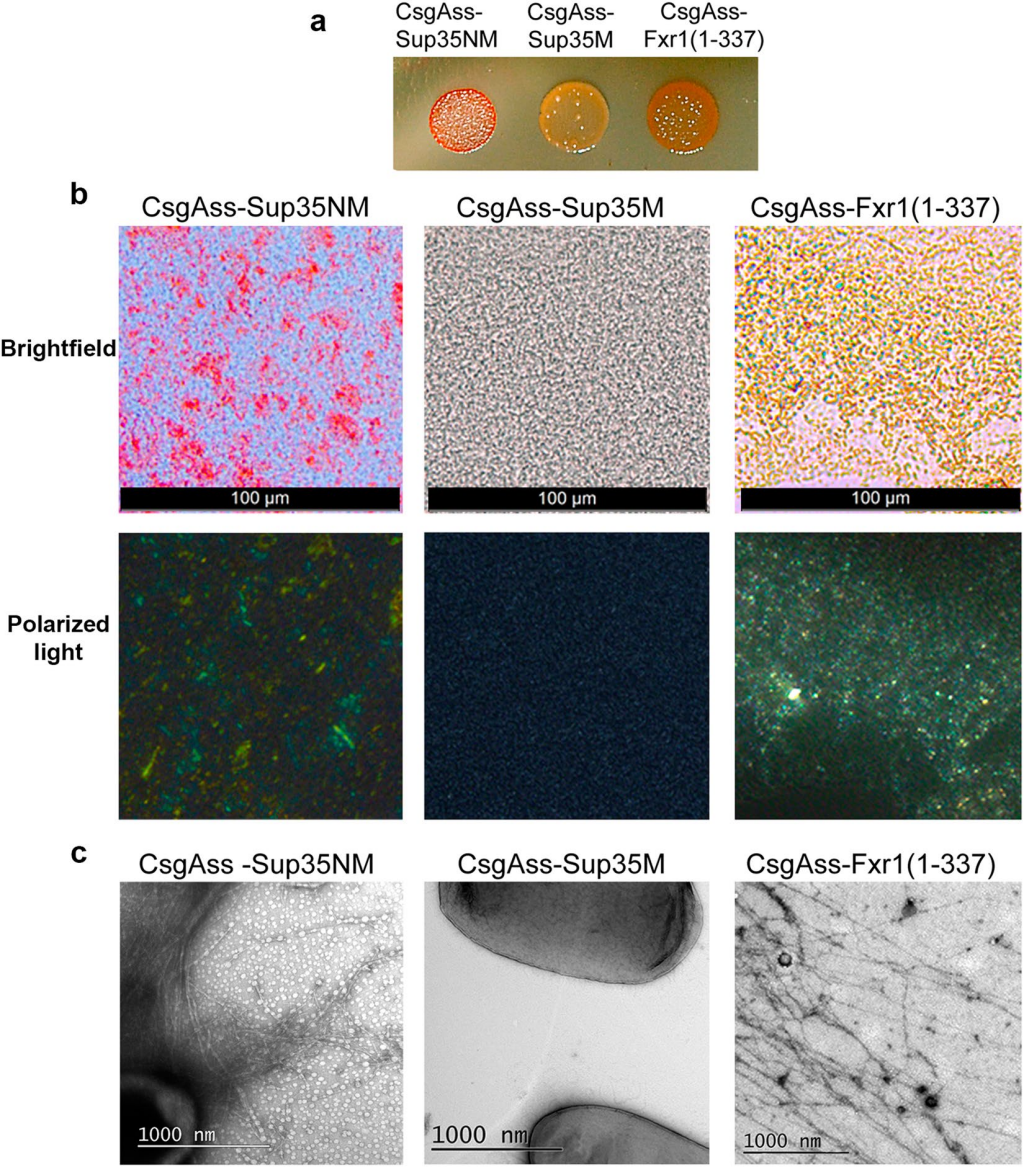
FXR1 (1-337) fragment demonstrates amyloid properties in the bacteria-based C-DAG system. (**a**) *E.coli* cells producing CsgAss-Sup35NM and CsgAss-FXR1(1-337) proteins form red colonies whereas cells producing CsgAss-Sup35M form pale colonies on agar plates containing CR. (**b**) Micrographs of CsgAss-Sup35NM, CsgAss-Sup35M and CsgAss-FXR1(1-337) scraped cell samples harvested from CR-containing agar. Extracellular material containing CsgAss-Sup35NM and CsgAss-FXR1(1-337) binds CR (upper panel) and displays yellow-green birefringence when viewed between crossed polarizers (lower panel). Scale bar, 100 μM (**c**) Secreted CsgAss-Sup35NM and CsgAss-FXR1(1-337) proteins form fibrils visualized by transmission electron microscopy. Scale bar, 1000 nM. The *E. coli* cells are seen as dark spots on the middle image.

To verify amyloid properties of the N-terminal fragment of FXR1, we also used the bacterial curli-dependent amyloid generator (C-DAG) system for production of extracellular amyloid fibrils^29,30^. This system relies on the ability of *E. coli* cells to generate surface associated amyloid fibrils (curli) composed of CsgA protein. This protein contains an N-terminal signal sequence (CsgA_SS_) that directs it to the cell surface and a C-terminal sequence that allows formation of extracellular fibrils^31,32^. Joining the signal sequence (CsgA_SS_) to heterologous amyloidogenic protein fragments directs their export to the cell surface where they can form fibrils. Since the C-DAG is applicable for the study of relatively short protein fragments^30^, we analyzed the amyloidogenic properties of the N-terminal fragment of FXR1 spanning the first 337 amino acids. The *E. coli* VS39 strain was transformed with the pVS-FXR1(1-337) plasmid providing expression of CsgA_SS_ fused to the FXR1(1-337) fragment. The VS39 cells expressing CsgA_SS_-Sup35NM and CsgA_SS_-Sup35M proteins were used as positive and negative controls, respectively. We showed that expression of CsgA_SS_-FXR1(1-337) resulted in red colonies of transformants, although the red coloration was not as intense as in transformants expressing CsgA_SS_-Sup35NM (Fig. 7a). The transformants expressing CsgA_SS_-Sup35M protein were pale on the medium containing Congo Red (Fig. 7a). CsgA_SS_-FXR1(1-337) and CsgA_SS_-Sup35NM proteins, but not CsgA_SS_-Sup35M, bound Congo Red and exhibited yellow-green birefringence when examined between crossed polarizers (Fig. 7b). This property is characteristic of amyloid fibrils^33^. Using transmission electron microscopy we detected the extracellular fibrils of the CsgA_SS_-FXR1(1-337) and CsgA_SS_-Sup35NM proteins, but not of the CsgA_SS_-Sup35M protein (Fig. 7c). Taken together, these data show that the N-terminal region of the FXR1 protein forms amyloid fibrils both *in vitro* and in the bacterial-based C-DAG system.

### Amyloid properties of the FXR1 protein are evolutionary conserved

**Figure 8:**
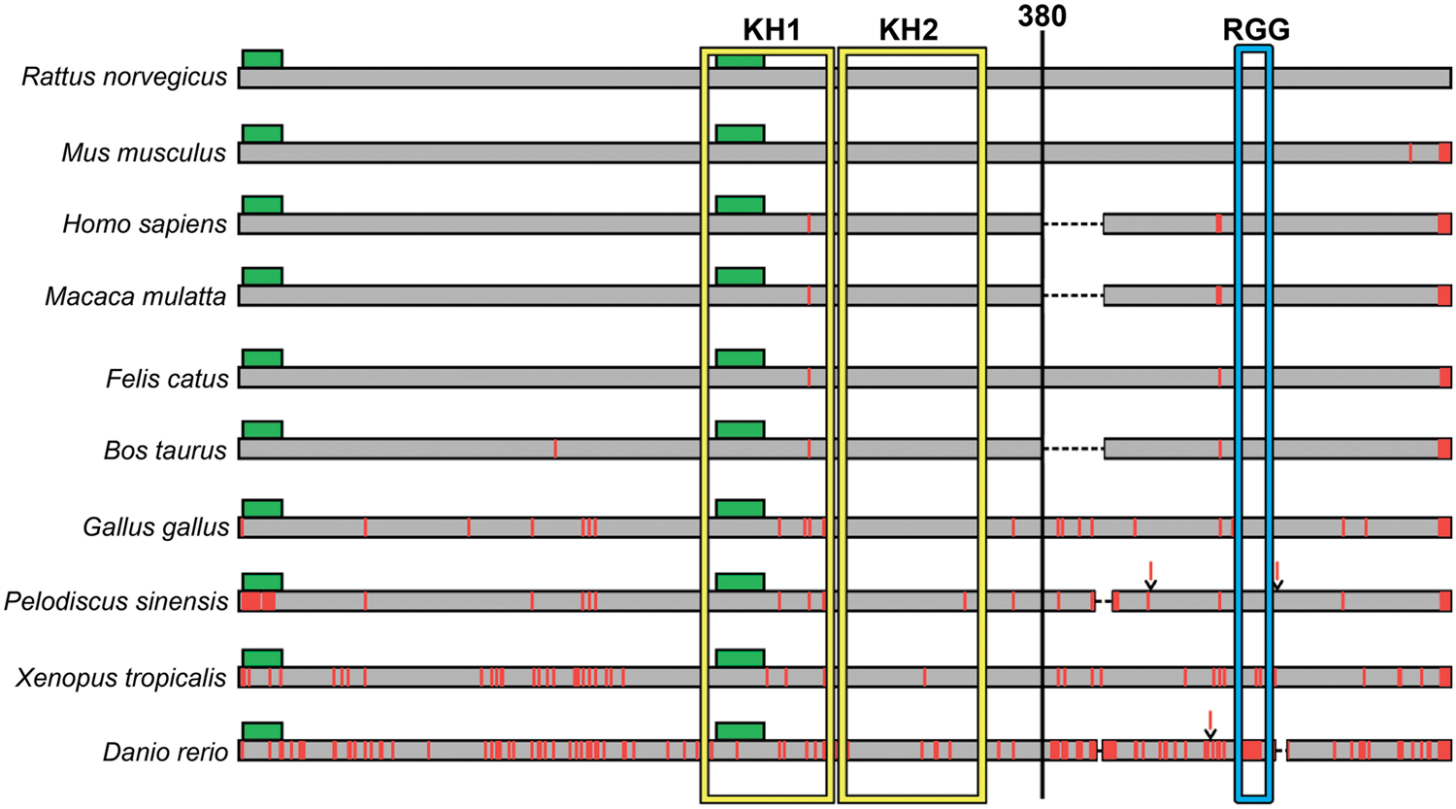
Diagram of the sequence alignment for the full-length FXR1 proteins from ten vertebrate species. The sequence of the FXR1 protein of Wistar rats is marked in gray. Potential amyloidogenic regions are marked as green rectangles. Positions of amino acids differing from those in the sequence of rat FXR1 are marked by vertical red lines. Insertions of amino acids are marked as red lines above the sequences. Deletions are indicated by a dashed black line. RNA binding domains KH1 and KH2 are enclosed in a yellow frame, and RGG domain – in a blue frame. See also Supplementary Fig. S8.

In order to analyze the evolutionary conservatism of FXR1, the ORF of corresponding gene was PCR amplified from the total cDNA obtained from brain-extracted mRNA of Wistar rats and sequenced (GenBank, accession number MG938503). The deduced amino acid sequence of the rat FXR1 protein was compared with canonical sequences of the FXR1 protein from different vertebrate species available in the UniProt database (http://www.uniprot.org). Since the amyloid properties of FXR1 depend on its N-terminal region, the comparative analysis was carried out separately for the N- and C-terminal regions of this protein. The N-terminal sequence contains KH1 and KH2 RNA-binding motives, whereas the C-terminal region contains RGG (G quartet RNA motif)^16^. The FXR1N(1-379 aa) sequences of rat and mouse are identical (Figs. 8 and S8). They differ from human, macaque and cat FXR1N sequences only by one amino acid – glutamic acid (E) in 270-th position is replaced by aspartic acid (D) (Supplementary Fig. S8). Both, E and D, belong to the class of the polar negatively charged amino acids, and this replacement is unlikely to change the amyloidogenic properties of the FXR1 protein. The N-terminal sequences of human, cat and macaque FXR1 proteins are identical. The FXR1N sequence of *Bos taurus* differs by two amino acids from the rat sequence (Figs. 8 and S8). The N-terminal regions of FXR1 proteins of non-mammalian vertebrates differ from the rat FXR1N more significantly, but all of them contain two identically arranged potentially amyloidogenic regions (Fig. 8). The C-terminal parts of FXR1 proteins of some species lack short regions before the RGG RNA-binding motive. No potentially amyloidogenic regions were revealed in FXR1C in all species analyzed. Thus, the N-terminal amyloid-forming region of FXR1 is highly conserved in the mammalian lineage and contains identically arranged amyloidogenic sequences in vertebrate species.

**Figure 9:**
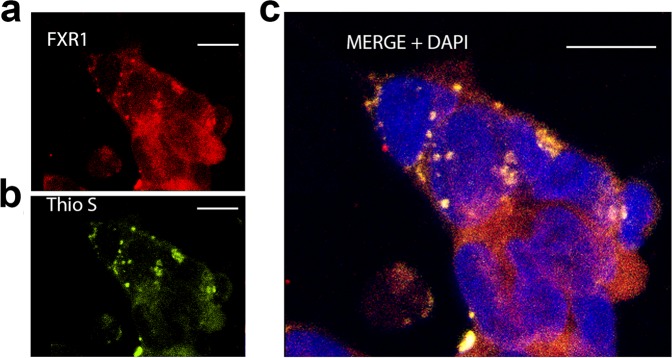
FXR1 colocalizes with Thioflavin S in neuroblastoma cells. IMR32 cells grown on coverslips were fixed, and immunostaining was performed to localize FXR1. FXR1 is present in the perinuclear cytoplasm of neuroblastoma cells (**a**) and colocalizes with amyloid-specific dye Thioflavin S (**b**,**c**). Scale bar for sections (**a**–**c**) is 10 µm. Over a hundred IMR32 cells were analyzed.

We tested the ability of the FXR1 protein to form amyloid aggregates in a human neuroblastoma cell culture. IMR32 cells grown on coverslips were fixed, and immunostaining was performed to localize FXR1. At the next step, the cells were stained with Thioflavin S. FXR1 forms both diffuse and granular structures around the nucleus of neuroblastoma cells (Fig. 9a,b). Most important, FXR1 colocalizes with the signals of Thioflavin S (Fig. 9c). These data support our hypothesis that the FXR1 protein is presented in amyloid form in neuronal cells.

## Discussion

Proteomic screening, based on the universal biochemical properties of amyloids, provides us with new opportunities for the study of the prevalence and role of amyloids in nature. Using an original proteomic approach, we identified a set of proteins forming amyloid-like aggregates in the rat brain. The data obtained by PSIA-LC-MALDI opens up broad prospects for further identification of functional amyloids in mammalian brain. We focused on the analysis of amyloid properties of the FXR1 protein, but at the same time we identified several other promising candidates for the role of functional amyloids. The NSF, MBP, RIMS1 and STXB1 proteins were identified by mass-spectrometry in the SDS-resistant fraction of all analyzed brain samples (Table 1; Supplementary Figs. S1–S5). All of them, except MBP, contain potentially amyloidogenic regions predicted by ArchCandy algorithm. The analysis of their amyloid properties will be the subject of further research. Notably, there are some data suggesting that the myelin basic protein (MBP), that is a component of specialized membrane covering axons, may normally form amyloid structures in the brain^21^. The CPEB3 protein was not identified in the SDS-resistant fraction in our proteomic screen. This is not surprising, since it was shown in the mouse model that CPEB3 aggregates only in the brains of specifically stimulated animals^8^.

The FRX1 protein identified by proteomic screening forms oligomers and large insoluble aggregates in rat brain. The monomers of FXR1 are almost undetectable (Fig. 1a). We showed that this protein, like other well-known amyloids, forms SDS-resistant conformers (Fig. 1c). Moreover, FXR1 clearly colocalizes with amyloid-specific dyes Congo Red, Thioflavin S and Thioflavin T on cryosections of rat cerebral cortex (Figs. 2a, 3a and S7). FXR1 extracted from brain by immunoprecipitation shows yellow-green birefringence after staining with Congo red (Fig. 2b). Taking together, all these data demonstrate that FXR1 presents in brain of healthy rats in the amyloid form. Functional role of amyloid conformers of FXR1 in mammalian brain remains to be elucidated. Previously it was shown that the FXR1 protein contains KH and RGG RNA-binding motives and differently regulates RNA stability and translation^17^. Changes in the expression level of this protein in brain cortex affect long-term memory and emotional state^14,15^. We demonstrated that amyloid conformers of FXR1 in neurons are colocalized with mRNA molecules resistant to RNAse treatment (Fig. 3c). Based on these data, we assume that the RNP particles containing FXR1 protect RNA from degradation. At the same time, cytological data do not allow to draw a final conclusion about protective function of FXR1 amyloid. We cannot exclude the possibility that other proteins interacting with FXR1 or with FXR1-bound mRNAs affect the stability of RNA as a part of the FXR1-containing RNP particles. It was previously shown that FXR1 may be involved in the stress granule formation in cell culture, but only under adverse conditions^34,35^. However, we analyzed brain cryosections of young healthy animals that were not exposed to stress. Taking into account the clear colocalization of FXR1 with a stable mRNA fraction in cortical neurons, the hypothesis that amyloid conformers of FXR1 perform protective functions seems to be the most justified. The protection of RNA mediated by amyloid-based RNP particles can be an important component of the regulation of gene expression in such long-living cells as neurons.

The N-terminal part of FXR1 contains algorithmically predicted potentially amyloidogenic regions (Table 1). We confirmed these amyloid properties of N-terminal fragment of FXR1 using a yeast-based system, *in vitro* experiments and the bacterial-based C-DAG system (Figs. 4–7). Moreover, N-terminal fragment of FXR1 is highly conserved across mammals and contains identically arranged potentially amyloidogenic sequences in evolutionary distant vertebrates (Figs. 8 and S8). These data suggest that amyloid properties of FXR1 are evolutionary conserved, especially in the mammalian lineage including humans. Our data obtained in human neuroblastoma cells (Fig. 9) confirm this hypothesis.

Taken together, we provide comprehensive evidence that the RNA-binding protein FXR1 is present in the amyloid form in the rat brain and its N-terminal amyloidogenic fragment is evolutionarily conserved. Our data open up prospects for studying the functional role of amyloids in the mammalian brain.

## Materials and Methods

### Animals

Six-month-old male Wistar rats were purchased in Rappolovo breeding colony (Saint-Petersburg, Russia). All animal experiments were conducted in accordance with the Treaty of Lisbon amending the Treaty on European Union and the Treaty establishing the European Community and entered into force on 1 December 2009. Experiments were approved by the Ethical Committee for Animal Research of St. Petersburg State University (conclusion # 131-03-6). Euthanasia of animals was carried out immediately after delivery to Saint-Petersburg State University.

### Proteomic screening and identification of proteins forming amyloid-like aggregates

Proteomic screening and identification of proteins forming amyloid-like aggregates in brain of *Rattus norvegicus* were performed using PSIA–LC–MALDI approach described recently^9^, with modifications. Brain homogenates were solubilized in Tris-buffered saline (TBS) (30 mM Tris-HCl, pH 7.4, 150 mM NaCl), supplemented with 10 mM PMSF and Complete Protease Inhibitor (Roche). The obtained lysates were fractionated by ultracentrifugation (151000 g, 2 h, 4 °C). Pellets containing protein aggregates were resuspended in TBS with 200 µg/ml RNase A (Thermo Fisher, USA), incubated for 15 min and treated with 1% SDS for 8 hours at 18 °C. Then, detergent-resistant protein complexes were separated by ultracentrifugation at 151000 g (8 h, 18 °C) through 25% sucrose-TBS cushion with 0.1% SDS. Pellets were suspended in water, sedimentated again at 151000 g (2 h, 4 °C) and denatured. After trypsinolysis the peptide mixtures were loaded (1 μl) onto an Acclaim PepMap 300 HPLC reverse-phase column (150 mm, 75 μm, particle size 5 μm; Thermo Scientific, USA) and separated in an acetonitrile gradient (2–90%) during 45 min using an UltiMate 3000 UHPLC RSLC nano high-performance nanoflow liquid chromatograph (Dionex, USA). Peptide fractions were collected every 10 s and loaded onto a 384-sample MTP AnchorChip 800/384 microtiter plate (Bruker Daltonics) using a Proteineer fc II spotter (Bruker Daltonics).

Peptides were identified using the Ultraflextreme MALDI-TOF/TOF mass spectrometer (Bruker Daltonics, DE). MS-spectra for each peptide fraction were determined and analyzed using WARP-LC software. MS/MS-analysis was performed for these peptides in fractions (spots) with maximal concentration (peak intensity) of these peptides. Match between the experimental spectra and corresponding proteins was analyzed using Mascot version 2.4.2 software (Matrix Science; http://www.matrixscience.com) in the UniProt database (http://www.uniprot.org/) restricted to *Rattus norvegicus*. α-cyano-4-hydroxycinnamic acid was used as a matrix. During analysis, preset parameters of “Mass tolerance” were used (precursor mass tolerance 100 ppm, fragment mass tolerance 0.9 Da). As a standard sample, Peptide Calibration Standard II 8222570 (Bruker Daltonics) was applied. Carboxymethylation of cysteine, partial oxidation of methionine, and one skipped trypsinolysis site were considered as permissible modifications. The BioTools software (Bruker, Bremen, Germany) was used for manual validation of protein identification.

### Rat brain slices and homogenization

For immunohistochemistry and fluorescent *in situ* hybridization rat brains were extracted, washed with PBS and fixed in 4% PFA for 3 hours. After fixation brains were embedded in FSC22 compound (Leica), frozen in liquid nitrogen and stored at −70 °C. Brains were sectioned to 20 µm thick slices on a freezing microtome CM1850UV (Leica). For PSIA–LC–MALDI analysis brains after extraction were immediately frozen in liquid nitrogen and homogenized using a cryogenic laboratory mill Freezer/Mill 6870 (SPEX SamplePrep).

### Immunohistochemistry, FISH, FRAP and immunoprecipitation

Immunohistochemistry and histological staining were performed on rat brain cryosections and IMR32 fixed cells. The primary Anti-FXR1 antibody ab129089 (1:200, Abcam, Cambridge, MA) and secondary antibody Goat anti-Rabbit IgG (H + L) conjugated with Alexa Fluor 546 or with Alexa Fluor 488 (1:500, ThermoFisher Scientific, USA) were used. The slides were also subjected to DAPI nuclear staining. Histological staining for amyloids was done using 0.1 mg/ml Congo red water solution. Staining for amyloids also was done using 1% Thioflavin S and Thioflavin T solutions for the rat brain cryosections and 0.05% Thioflavin S solution for the IMR32 cells. The polyA mRNA on rat brain cryosections was detected with biotinylated oligo(dT)20 (Beagle, Russia) followed by avidin conjugated with AlexaFluor488 (1:200, ThermoFisher Scientific, USA). Enzymatic treatment was performed with RNaseA (500 µg/ml, ThermoFisher Scientific, USA) for one hour at 37 °C. The analysis of colocalization of the fluorescent labeled antibodies for the FXR1 protein with the amyloid specific dyes or with polyA mRNA was performed using a TCS SP5 confocal microscope (Leica Microsystems, Germany) and “Leica Application Suite X 3.3.0.16799” software. The colocalization of FXR1 with Congo red and Thioflavin S was analyzed by Pearson correlation analysis using the coloc2 plugin of FIJI software (http://fiji.sc/Fiji).

FRAP experiments using living *S. cerevisiae* cells expressing YFP-tagged proteins were acquired on a Leica TCS SP5 confocal microscope and were performed as described earlier^36^. A bleach region was bleached by ten iterations of 100% laser power. Five initial pre-bleach images and 25 subsequent post-bleach images were acquired with an interval of 30 seconds, and normalized fluorescence recovery was analyzed using FIJI software (http://fiji.sc/Fiji).

For immunoprecipitation of FXR1 from rat brain the anti-FXR1 antibodies ab129089 (Abcam, Cambridge, MA) were bound to protein A coated SileksMagX-Protein A magnetic beads (Sileks, Moscow, Russia) and incubated with *R. norvegicus* brain lysate with addition of Complete Protease Inhibitor (Roche) (+4 °C, overnight). Protein elution was performed in glycine buffer according to manufacturer’s protocol (Sileks, Moscow, Russia). Then the sample was concentrated by centrifugation at 130 000 g for 2 h, stained with Congo Red dye and analysed by brightfield, polarization and TEM microscopy.

### Cloning of the *FXR1* gene fragments

Total rat RNA was extracted from brain homogenates using TRIzol reagent (Invitrogen) according to the manufacturer protocol. cDNA synthesis was performed with SuperScript III Reverse Transcriptase (Invitrogen). cDNA was further used for *FXR1* fragments synthesis. The fragment of *FXR1* gene coding for 1-379 aa for expression in *E. coli* was amplified with *fxr1EcoRIforward* and *fxr1(379)BamHIreverse* primers and inserted into pET302 vector to obtain the pET302-FXR1(1-379) plasmid. The fragment of *FXR1* gene coding for 1-379 aa for expression in *S. cerevisiae* was amplified with *fxr1HindIIIforward* and *fxr1(YFP)BamHIreverse* primers. The *fxr1(380)HindIIIforward* and *fxr1(568)BamHIreverse* primers were used for amplification of the fragment of *FXR1* gene coding for 380-568 aa. Both fragments were inserted in pCUP-YFP vector to obtain pCUP-FXR1(1-379)-YFP and pCUP-FXR1(380-568)-YFP plasmids. The fragment of the *FXR1* gene coding for 1-337 aa for expression in C-DAG system was amplified with *fxr1NotIforward* and *fxr1XbaIreverse* primers and this fragment was inserted into pVS72 vector to obtain the pVS-FXR1(1-337) plasmid. All primers are listed in SupplementaryTable S1.

### Recombinant protein expression and purification

FXR1(1-379) was expressed as 6His- tagged fusion protein in Rosetta (DE3) pLysS cells (Novagen) in LB medium. Cells were lysed in 0.05 M Tris (pH 7.6), 0.15 M NaCl and complete protease inhibitor cocktail (Roche) by ultrasonication. Inclusion bodies were dissolved in 8 M urea, 20 mM Na phosphate, 0,25 M NaCl, 5 mM imidazole and 1 mM βME. The cleared lysate was loaded onto a gravity NiNTA column (Qiagen), washed with 8 M urea, 20 mM Na phosphate, 0,25 M NaCl, 5 mM imidazole and 1 mM β-mercaptoethanol, and eluted in 8 M urea, 20 mM Na phosphate, 0,5 M NaCl, 500 mM imidazole and 1 mM βME. The purity of the recombinant protein was verified by SDS-PAGE, protein concentration was measured using Qubit 2 Fluorometer (Thermo Scientific).

### *In vitro* fibril formation assay

The experiment was performed as described previously^37^. In brief, purified FXR1(1-379) protein solution diluted to 5 μM in a buffer containing 20 mM Tris (pH 8.0), 1 mM βME was incubated at 37 °C under agitation for 48 hr with slow rotation. Fibril formation was verified by TEM and Congo Red staining.

### Electron microscopy

TEM images were recorded on a Jeol JEM-2100 microscope. Negatively-stained samples were prepared on formvar film 300 mesh copper grids (Electron Microscopy Sciences). A 10 μl aliquot of fibril solution (from *in vitro* experiment) or bacterial culture suspension (from C-DAG experiment) were adsorbed to the formvar film for one minute, blotted, washed twice with 10 μl of water for 10 s, stained with 10 μl of 1% uranyl acetate for 1 minute and dried in air.

### Congo red staining of fibrils

10 µl of the aggregated protein solution was put onto a glass microscope slide, air-dried, stained with 50 µl of Congo Red water solution (2,5 mg/ml), washed with water and analyzed in brightfield and between cross polarizers on the inverted microscope Leica DMI6000 B. Images were acquired using the Leica Application Suite software.

### Protein analysis

Preparation of protein lysates was performed as described previously^38^ with addition of RNase A (Thermo Fisher, USA) to the lysis buffer to final concentration 200 µg/ml. Rat brain lysates were fractionated for 25 min at 9800 g, +4 °С, and the insoluble fraction was collected. The supernatant was loaded on the Amicon Ultra 100 K filter (Merck, Millipore) and further divided into two fractions: less than 100 kDa and more than 100 kDa. The FXR1 protein was detected with the primary Anti-FXR1 antibody ab129089 (Abcam) and secondary Goat Anti-Rabbit IgG H&L (ab205718) (HRP) (Abcam). Chemiluminescent detection was performed using the Amersham ECL Prime Western Blotting Detection Reagent (GE Healthcare, USA).

Fractionation of yeast cell lysates was performed according to^39^. The cells were harvested, washed in water, and homogenized by glass beads in a buffer A: 25 mM Tris-HCl, pH 7.4, 100 mM NaCl, 1 mM dithiothreitol. To prevent proteolytic degradation, 10 mM EDTA, 2 mM PMSF, and Complete™ protease inhibitor mixture (Roche Applied Science) were added. Cell lysate was centrifuged for 25 min at 9800 g, +4 °С, then supernatant was transferred to a new tube and debris was suspended in equal buffer volume. Semi-denaturing detergent agarose gel electrophoresis (SDD-AGE)^11,23^ was performed using 1% agarose gel. Before loading onto a gel, protein extracts were treated for 10 min with 1% SDS at room temperature. Proteins fused with YFP were detected with polyclonal chicken primary antibodies against GFP (ab13970, Abcam, Great Britain) and secondary Goat Anti-Chicken IgY H&L (HRP) (ab6877, Abcam). Chemiluminescent detection was performed using the Amersham ECL Prime Western Blotting Detection Reagent (GE Healthcare, USA).

### Yeast strains and growth conditions

The *S. cerevisiae* strain BY4742^40^ [*MATα his3Δ1 leu2Δ0 lys2Δ0 ura3Δ0*] was used for analysis of the FXR1 fragments aggregation. Standard yeast genetic techniques, media, and cultivation conditions were used. 150 μM copper sulfate (CuSO_4_) was added to synthetic medium to induce the expression of genes under the *P*_*CUP1*_ promoter.

### Analysis of amyloid fibril formation in the bacteria-based system C-DAG

The analysis of amyloid fibril formation of FXR1(1-337aa) fragment in the bacteria-based C-DAG system was performed as described earlier^29,30^. *E. coli* strain VS39 was transformed with the plasmid pVS-FXR1(1-337) coding for FXR1(1-337) protein fused to CsgA signal sequence. VS39 transformants with pVS72 and pVS105 encoding the CsgA_SS_-Sup35NM and CsgA_SS_-Sup35M proteins were used as positive and negative controls of amyloid generation, respectively.

## Supplementary information

Supplementary Figures and Table

## Data Availability

All data generated and analyzed during this study are available from the corresponding author on reasonable request.
