# Supplementary material for: RNA-binding protein FXR1 is presented in rat brain in amyloid form
Source: Sci Rep. 2019 Dec 12;9:18983. doi: 10.1038/s41598-019-55528-6 (PMC6908614; doi:10.1038/s41598-019-55528-6)
Supplement: Supplementary file 1 — Supplementary Figures and Table [file 41598_2019_55528_MOESM1_ESM.pdf]

RNA-binding protein FXR1 is presented in rat brain in amyloid form

Julia V. Sopova, Elena I. Koshel, Tatiana A. Belashova, Sergey P. Zadorsky, Alexandra V. Sergeeva, Vera A. Siniukova, Alexandr A. Shenfeld, Maria E. Velizhanina, Kirill V. Volkov, Anton A. Nizhnikov, Daniel V. Kachkin, Elena R. Gaginskaya, Alexey P. Galkin

NSF Vesicle-fusing ATPase OS=Rattus norvegicus GN=Nsf PE=1 SV=1 NSF_RAT MH+(mono):1.008 MH+ (avg): 1.008

Tolerance (Da):0.500 Number of Peaks:775

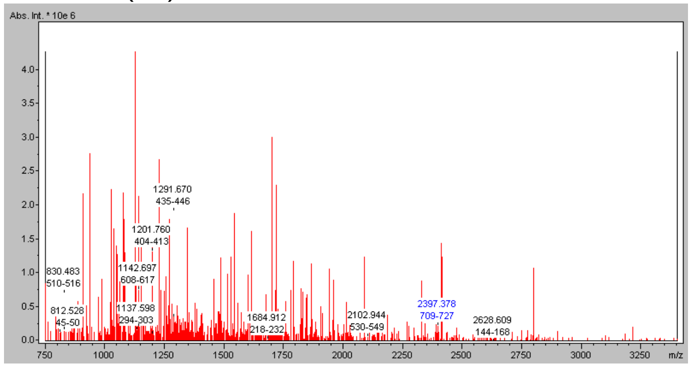

Tolerance (Da) 0.900 Number of Peaks: 1726

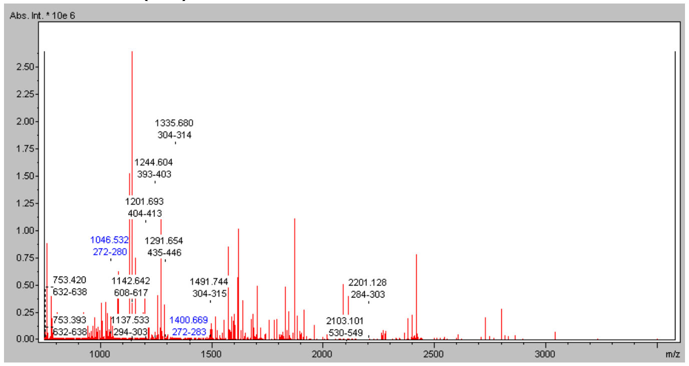

Tolerance (Da):0.500 Number of Peaks: 616

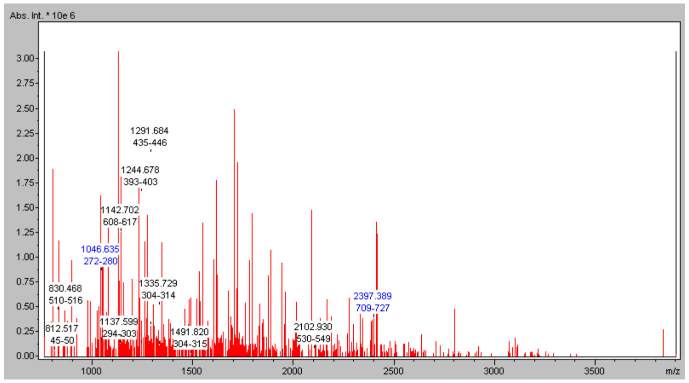

1 10 20 30 40 50 60
MAGRTMQAAR CPTDELSLSN CAVVNEKDYQ SGQHVMVRTS PNHKYIFTLR THPSVVPGCI
70 80 90 100 110 120
AFSLPQRKWA GLSIGQDIEV ALYSFDKAKQ CIGTMTIEID FLQKKNIDSN PYDTDKMAAE
130 140 150 160 170 180
FIQQFNHQAF SVGQQLVFSF NDKLFGLLVK DIEAMDPSIL KGEPASGKRQ KIEVGLVVGN
190 200 210 220 230 240
SQVAFEKAEN SSLNLIGKAK TKENRQSIIN PDWNFEKMGI GGLDKEFSDI FRRAFASRVF
250 260 270 280 290 300
PPEIVEQMGC KHVKGILLYG PPGCGKTLLA RQIGKMLNAR EPKVVNGPEI LNKYVGESEA
310 320 330 340 350 360
NIRKLFADAE EEQRRLGANS GLHIIIFDEI DAICKQRGSM AGSTGVHDTV VNQLLSKIDG
370 380 390 400 410 420
VEQLNNILVI GMTNRPDLID EALLRPGRLE VKMEIGLPDE KGRLQILHIH TARMRGHQLL
430 440 450 460 470 480
SADVDIKELA VETKNFSGAE LEGLVRAAQS TAMNRHIKAS TKVEVDMEKA ESLQVTRGDF
490 500 510 520 530 540 550 560 570 580 590
LASLENDIKP AFGTNQEDYA SYIMNGIIKW GDPVTRVLDD GELLVQQTKN SDRTPLVSVL LEGPPHSGKT ALAAKIAEES NFPFIKICSP DKMIGFSETA KCQAMKKIFD
600 610 620 630 640 650 660 670 680 690 700
DAYKSQLSCV VVDDIERLLD YVPIGPRFSN LVLQALLVLL KKAPPQGRKL LIIGTTSRKD VLQEMEMLNA FSTTIHVPNI ATGEQLLEAL ELLGNFKDKE RTTIAQQVKG
710 720 730 740
KKVWIGIKKL LMLIEMSLQM DPEYRVRKFL ALMREEGASP LDFD

Supplementary Figure S1. Mass spectrometry identification data of the NSF protein (see also Table 1) in three brain samples of six-month-old male rats. The amino acid sequence of the NSF protein is shown. The peptides identified by mass spectrometry are indicated in yellow.

MBP Myelin basic protein OS=Rattus norvegicus GN=Mbp PE=1 SV=3 MBP_RAT MH+ (mono):1.008 MH+ (avg): 1.008

Tolerance (Da):0.500 Number of Peaks:775

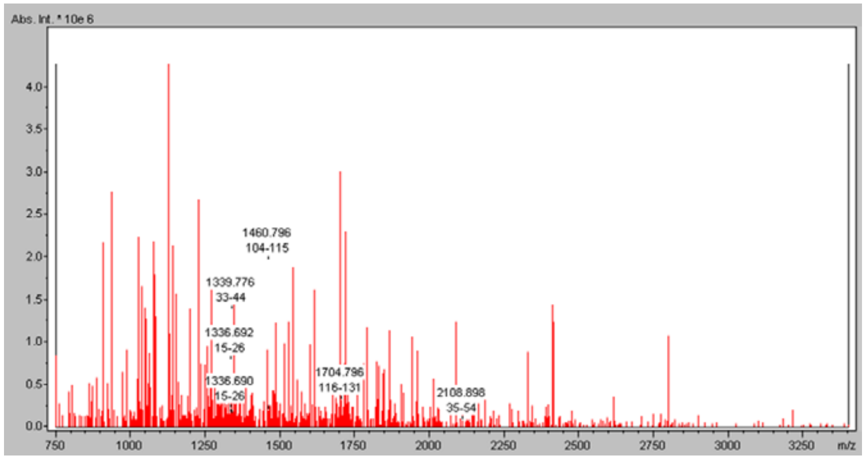

Tolerance (Da) 0.900 Number of Peaks: 1726

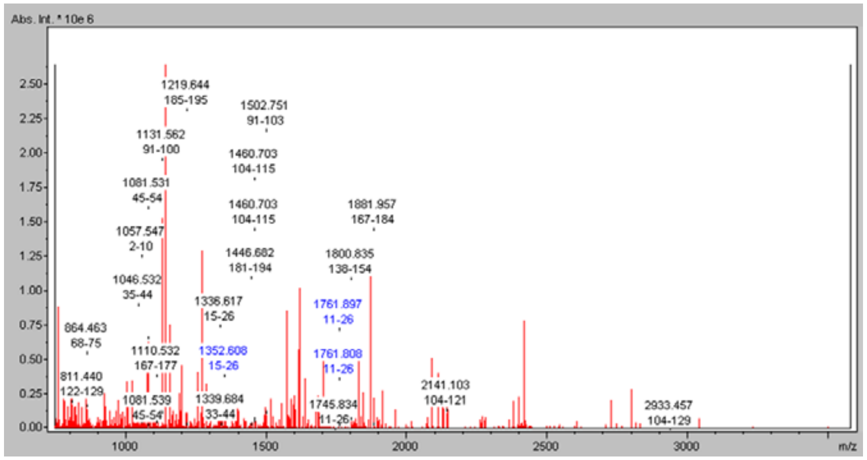

Tolerance (Da):0.500 Number of Peaks: 616

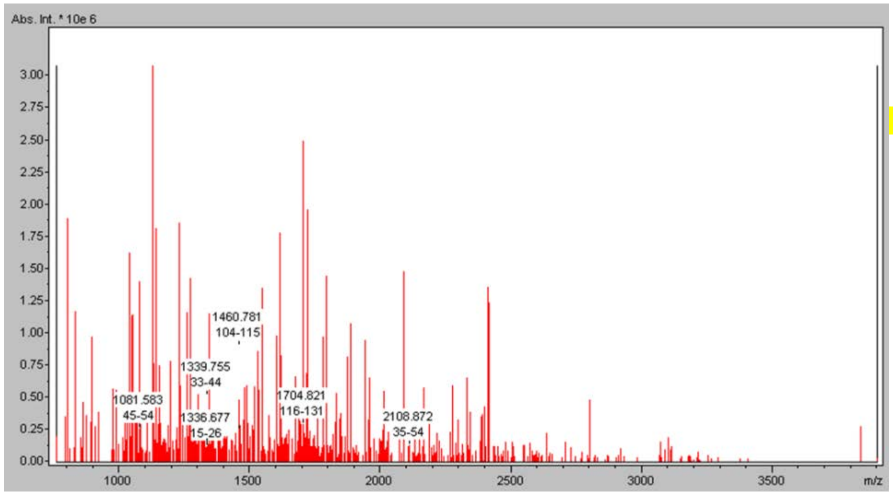

1 10 20 30 40 50
MASQKRPSQR HGSKYLATAS TMDHARHGFL PRHRDTGILD SIGRFFSGDR
60 70 80 90 100
GAPKRGSGKV PWLKQSRSPL PSHARSRPGL CHMYKDSHTR TTHYGSLPQK
110 120 130 140 150
SQRTQDENPV VHFFKNIVTP RTPPPSQGKG RGLSLSRFSW GAEGQKPGFG
160 170 180 190
YGGRASDYKS AHKGFKGAYD AQGTLSKIFK LGGRDSRSGS PMARR

Supplementary Figure S2. Mass spectrometry identification data of the MBP protein (see also Table 1) in three brain samples of six-month-old male rats. The amino acid sequence of the MBP protein is shown. The peptides identified by mass spectrometry are indicated in yellow.

RIMS1 Regulating synaptic membrane exocytosis protein 1 OS=*Rattus norvegicus* GN=Rims1 PE=1 SV=1 RIMS1_RAT

MH+ (mono):1.008   MH+ (avg): 1.008

Tolerance (Da):0.500 Number of Peaks:775

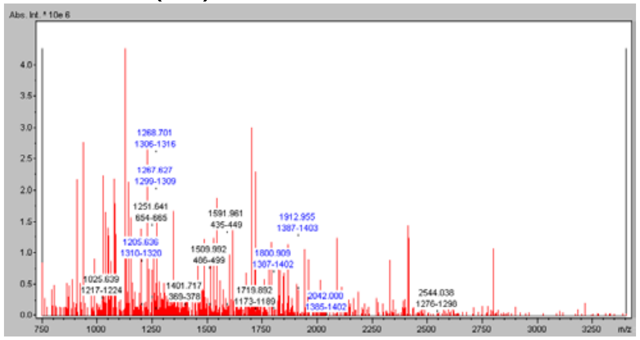

Tolerance (Da) 0.900 Number of Peaks: 1726

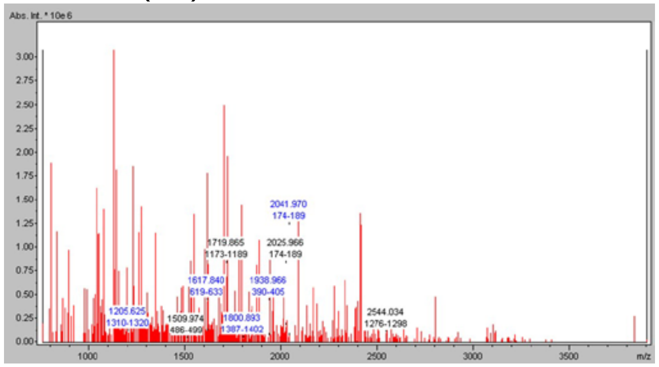

Tolerance (Da):0.500 Number of Peaks: 616

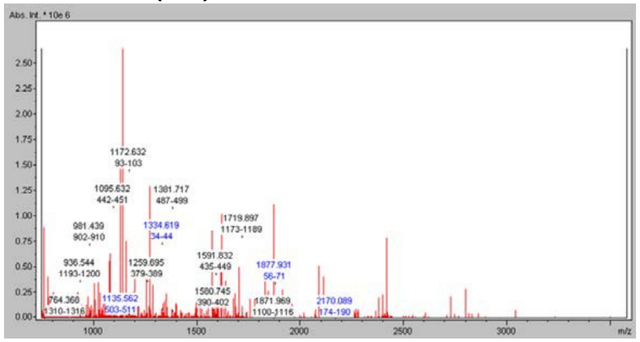

1 10 20 30 40 50 60 70 80
MSSAVGPRGP RPPTVPPPMQ ELPDLSHLTE EERNIIMAVM DRQKEEEEKE EAMLKCVVRD MAKPAACKTP RNAESQPHQP
90 100 110 120 130 140 150 160
PLNIFRCVCV PRKPSSEEGG PERDWRLHQQ FESYKEQVRK IGEEARRYQG EHKDDAPTCG ICHKTKFADG CGHLCSYCRT
170 180 190 200 210 220 230 240
KFCARCGGRV SLRSNNEDKV VMWVCNLCRK QQEILTKSGA WFFGSGPQQP SQDGTLSDTA TGAGSEVPRE KKARLQERSR
250 260 270 280 290 300 310 320
SQTPLSTAAV SSQDTATPGA PLHRNKGAEP SQQALGPEQK QASRSRSEPP RERKKAPGLS EQNGKGGQKS ERKRVPKSVV
330 340 350 360 370 380 390 400
QPGEGIADER ERKERRETRR LEKGRSQDYS DRPEKRDNGR VAEDQKQRKE EEYQTRYRSD PNLARYPVKA PPEEQQMRMH
410 420 430 440 450 460 470 480
ARVSRARHER RHSDVALPHT EAAAAAPAEA TAGKRAPATA RVSPPESPRA RAAAAQPPTE HGPPPPRPAP GPAEPPEPRV
490 500 510 520 530 540 550 560
PEPLRKQGRL DPGSAVLLRK AKREKAESML RNDSLSSDQS ESVRPSPPKP HRPKRGGKRR QMSVSSSEEE GVSTPEYTSC
570 580 590 600 610 620 630 640
EDVELESESV SEKGDLDYYW LDPATWHSRE TSPISSHPVT WQPSKEGDRL IGRVILNKRT TMPKESGALL GLKVVGGKMT
650 660 670 680 690 700 710 720 730 740 750 760 770 780
DLGRLGAFIT KVKKGSLADV VGHLRAGDEV LEWNGKPLPG ATNEEVYNII LESKSEPQVE IIVSRPIGDI PRIPESSHPP LESSSSSFES QKMERPSISV ISPTSPGALK DAPQVLPGQL SVKLWYDKVG HQLIVNVLQA
790 800 810 820 830 840 850 860 870 880 890 900 910 920
TDLPPRVDGR PRNPYVKMYF LPDRSDKSKR RTKTVKKLLE PKWNQTFVYS HVHRRDFRER MLEITVWDQP RVQDEESEFL GEILIELETA LLDDEPHWYK LQTHDESSLP LPQPSPFMPR RHIHGESSSK KLQRSQRISD
930 940 950 960 970 980 990 1000 1010 1020 1030 1040 1050 1060
SDISDYEVDD GIGVVPPVGY RASARESKAT TLTVPEQQRT THHRSRSVSP HRGDDQGRPR SRLPNVPLQR SLDEIHPTRR SRSPTRHHDA SRSPADHRSR HVESQYSSEP DSELLMLPRA KRGRSAESLH MTSELQPSLD
1070 1080 1090 1100 1110 1120 1130 1140 1150 1160 1170 1180 1190 1200
RARSASTNCL RPDTSLHSPE RERHSRKSER CSIQKQSRKG TASDADRVLP PCLSRRGYAT PRATDQPVVR GKYPTRSRSS EHSSVRTLCS MHHLAPGGSA PPSPLLLTRT HRQGSPTQSP PADTSFGSRR GRQLPQVPVR
1210 1220 1230 1240 1250 1260 1270 1280 1290 1300 1310 1320 1330 1340
SGSIEQASLV VEERTRQMKV KVHRFKQTTG SGSSQELDHE QYSKYNIHKD QYRSCDNASA KSSDSDVSDV SAISRASSTS RLSSTSFMSE QSERPRGRIS SFTPKMQGRR MGTSGRAIIK STSVSGEIYT LERNDGSQSD
1350 1360 1370 1380 1390 1400 1410 1420 1430 1440 1450 1460 1470 1480
TAVGTVGAGG KKRRSSLSAK VVAIVSRRSR STSQLSQTES GHKKLKSTIQ RSTETGMAAE MRKMVRQPSR ESTDGSINSY SSEGNLIFPG VRVGPDSQFS DFLDGLGPAQ LVGRQTLATP AMGDIQIGME DKKGQLEVEV
1490 1500 1510 1520 1530 1540 1550 1560 1570 1580 1590 1600 1610
IRARSLTQKP GSKSTPAPYV KVYLLENGAC IAKKKTRIAR KTLDPLYQQS LVFDESPQGK VLQVIVWGDY GRMDHKCFMG VAQILLEELD LSSMVIGWYK LFPPSSLVDP TLAPLTRRAS QSSLESSSGP PCIRS

Supplementary Figure S3. Mass spectrometry identification data of the RIMS1 protein (see also Table 1) in three brain samples of six-month-old male rats. The amino acid sequence of the RIMS1 protein is shown. The peptides identified by mass spectrometry are indicated in yellow.

STXBP1 Syntaxin-binding protein 1 OS=Rattus norvegicus GN=Stxbp1 PE=1 SV=1 STXB1_RAT MH+ (mono):1.008 MH+ (avg): 1.008

Tolerance (Da):0.500 Number of Peaks:775

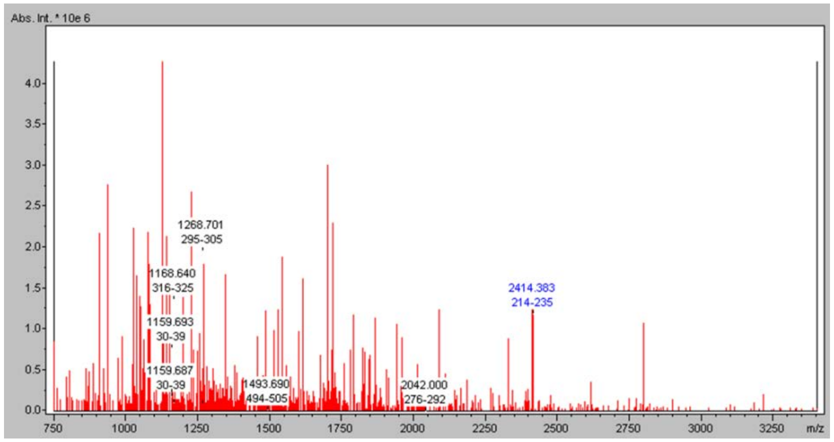

Tolerance (Da) 0.900 Number of Peaks: 1726

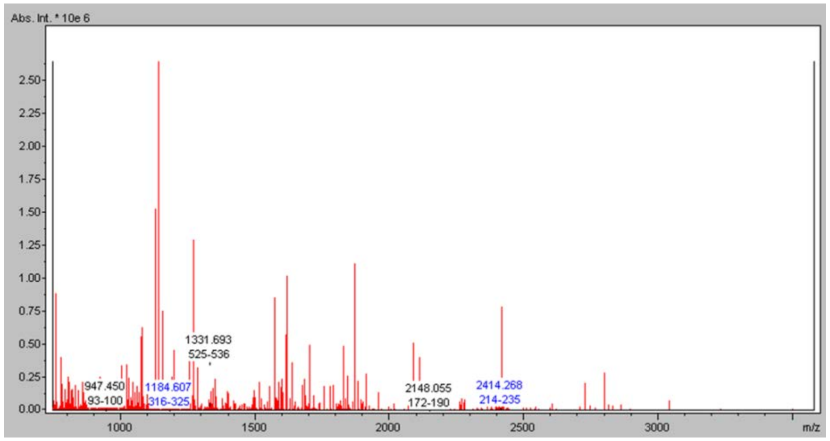

Tolerance (Da):0.500 Number of Peaks: 616

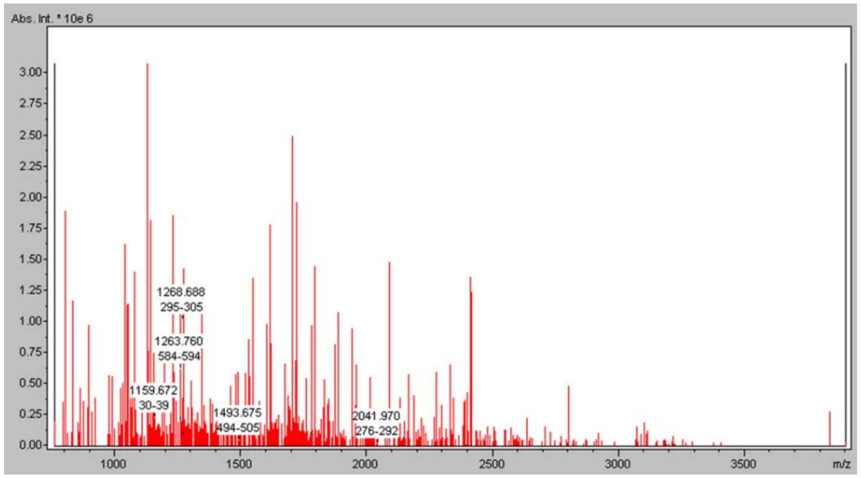

1 10 20 30 40 50 60 70
MAPIGLKAVV GEKIMHDVIK KVKKKGEWKV LVVDQLSMRM LSSCCKMTDI MTEGITIVED INKRREPLPS
80 90 100 110 120 130 140
LEAVYLITPS EKSVHSLISD FKDPPTAKYR AAHVFFTDSC PDALFNELVK SRAAKVIKTL TEINIAFLPY
150 160 170 180 190 200 210
ESQVYSLDSA DSFQSFYSPH KAQMKNPILE RLAEQIATLC ATLKEYPAVR YRGEYKDNAL LAQLIQDKLD
220 230 240 250 260 270 280
AYKADDPTMG EGPDKARSQL LILDRGFDPS SPVLHELTFQ AMSYDLLPIE NDVYKYETSG IGEARVKEVL
290 300 310 320 330 340 350
LDEDDDLWIA LRHKHIAEVS QEVTRSLKDF SSSKRMNTGE KTTMRDLSQM LKKMPQYQKE LSKYSTHLHL
360 370 380 390 400 410 420
AEDCMKHYQG TVDKLCRVEQ DLAMGTDAEG EKIKDPMRAI VPILLDANVS TYDKIRIILL YIFLKNGITE
430 440 450 460 470 480 490
ENLNKLIQHA QIPPEDSEII TNMAHLGVPI VTDSTLRRRS KPERKERISE QTYQLSRWTP IIKDIMEDTI
500 510 520 530 540 550 560
EDKLDTKHYP YISTRSSASF STTAVSARYG HWHKNKAPGE YRSGPRLIIF ILGGVSLNEM RCAYEVTQAN
570 580 590
GKWEVLIGST HILTPQKLLD TLKKLNKTDE EISS

Supplementary Figure S4. Mass spectrometry identification data of the STXBP1 protein (see also Table 1) in three brain samples of six-month-old male rats. The amino acid sequence of the STXBP1 protein is shown. The peptides identified by mass spectrometry are indicated in yellow.

FXR1 Fragile X mental retardation syndrome-related protein 1 OS=*Rattus norvegicus* GN=Fxr1 PE=1 SV=1 FXR1_RAT
MH+ (mono):1.008   MH+ (avg): 1.008

Tolerance (Da):0.500 Number of Peaks:775

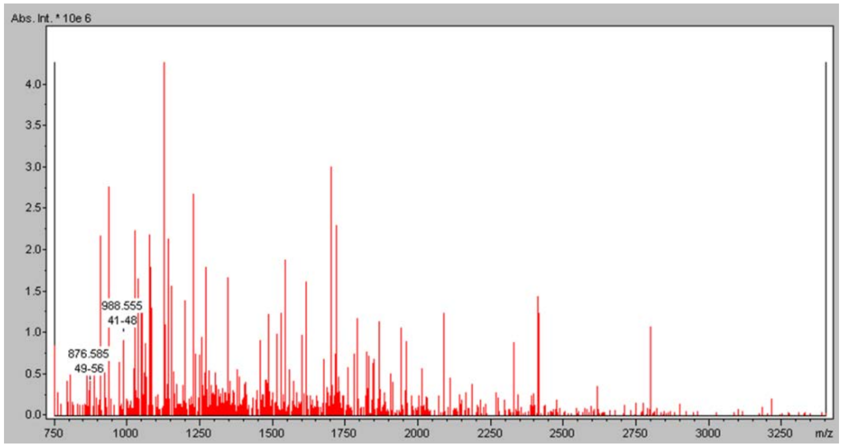

Tolerance (Da) 0.900   Number of Peaks: 1726

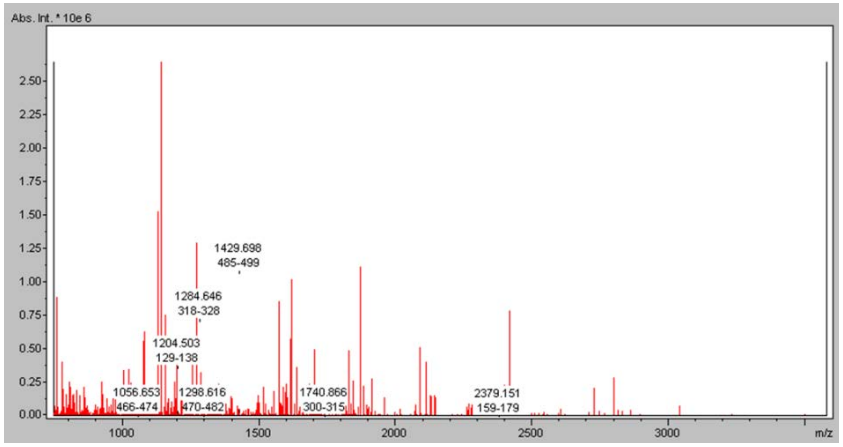

Tolerance (Da):0.500 Number of Peaks: 616

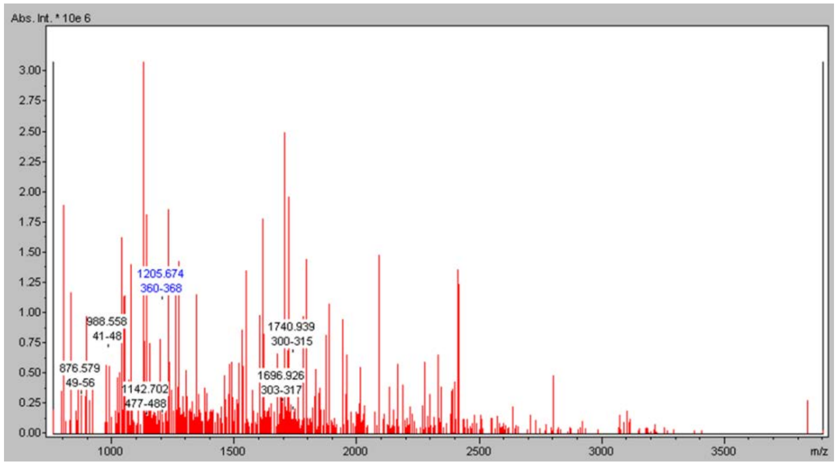

10 20 30 40 50 60 70
MAELTVEVRG SNGAFYKVFI KDVHEDSLTV VFENNWQPER QVPFNEVRLP PPPDIKKEIS EGDEVEVYSR
80 90 100 110 120 130 140
ANDQEPCGWW LAKVRMMKGE FYVIEYAACD ATYNEIVTFE RLRPVNQNKT VKKNTFFKCT VDVPEDLREA
150 160 170 180 190 200 210
CANENAHKDF KKAVGACRIF YHPETTQLMI LSASEATVKR VNILSDMHLR SIRTKLMLMS RNEEATKHLE
220 230 240 250 260 270 280
CTKQLAAAFH EEFVVREDLM GLAIGTHGSN IQQARKVPGV TAIELDEDTG TFRIYGESAE AVKKARGFLE
290 300 310 320 330 340 350
FVEDFIQVPR NLVGKVIGKN GKVIQEIVDK SGVVRVRIEG DNENKLPRED GMVPFVFVGT KESIGNVQVL
360 370 380 390 400 410 420
LEYHIAYLKE VEQLRMERLQ IDEQLRQIGM GFRPSSTRGP EKEKGYATDE STVSSVQGSR SYSGRGRGRR
430 440 450 460 470 480 490
GPNYTSGYGT NSELSNPSET ESERKDELSD WSLAGEDDRE TRHQRDSRRR PGGRGRSVSG GRGRGGPRGG
500 510 520 530 540 550 560
KSSISSVLKD PDSNPYSLLD NTESDQTADT DASESHHSTN RRRRSRRRRT DEDAVLMDGM TESDTASVNE
568
NGLGKRCD

Supplementary Figure S5. **Mass spectrometry identification data of the FXR1 protein (see also Table 1, Figure 1) in three brain samples of six-month-old male rats.** The amino acid sequence of the FXR1 protein is shown. The peptides identified by mass spectrometry are indicated in yellow.

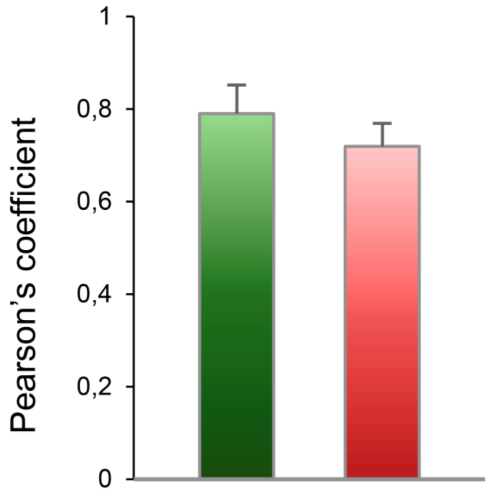

Supplementary Figure S6. Pearson correlation coefficients for Fxr1 and Thioflavin S (green) and for Fxr1 and Congo red (red). Colocalization of Fxr1 and amyloid specific dyes in 100 cells for each variant was analyzed by Pearson correlation analysis using the coloc2 plug-in of FIJI. Mean value of Pearson correlation coefficient for Fxr1 and Thioflavin S staining was 0,79±0,06; for Fxr1 and Congo red staining was 0,72±0,05.

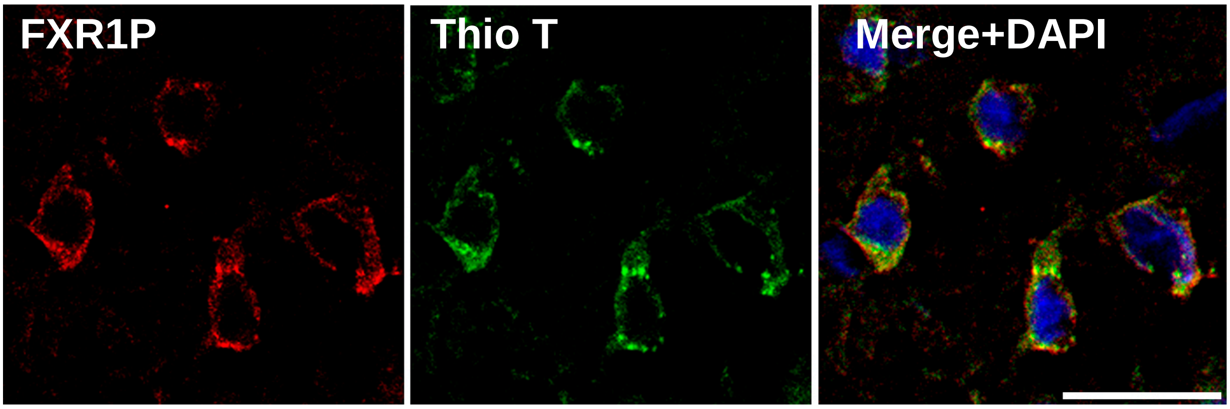

Supplementary Figure S7. FXR1 colocalizes with Thioflavin T in cortical neurons. FXR1 is present in the perinuclear cytoplasm of cortical neurons and colocalizes with amyloid-specific dye Thioflavin T. Scale bar is 20 μm

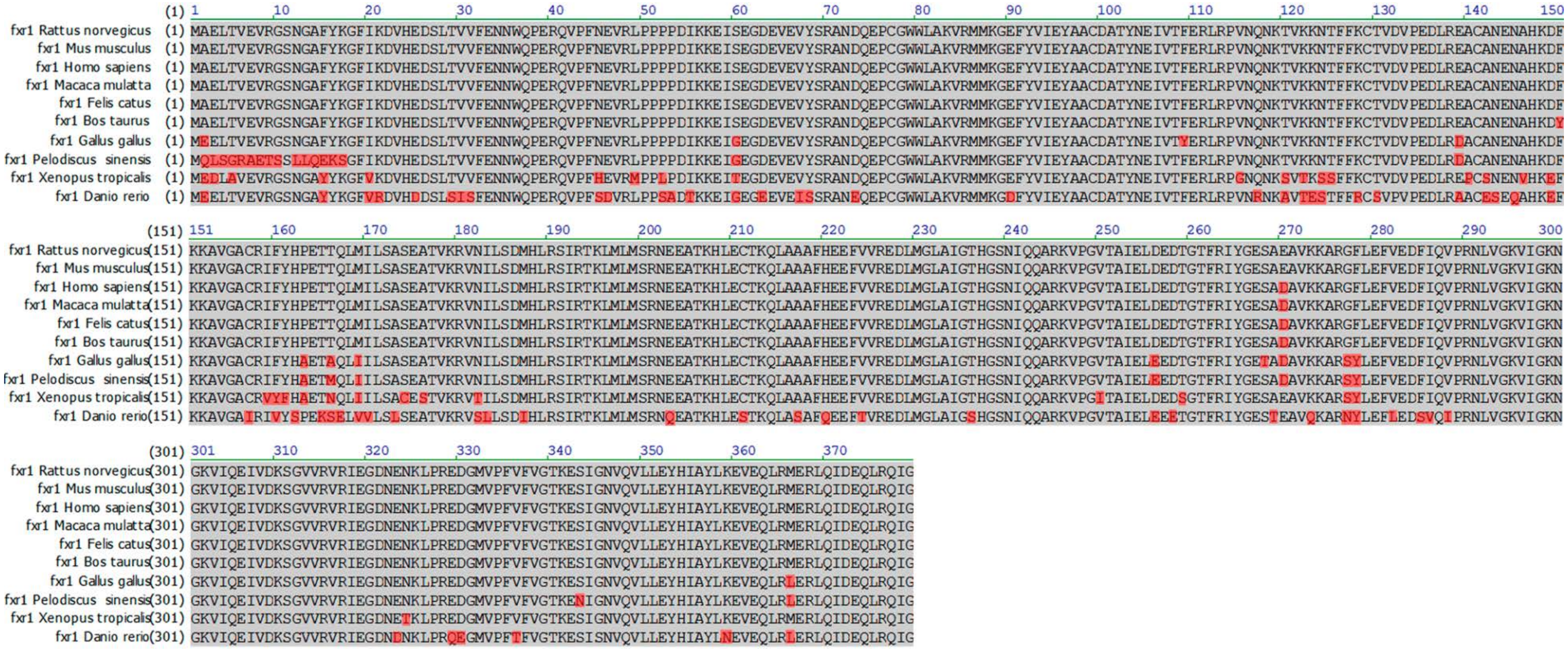

Supplementary Figure S8. N-terminal amyloidogenic region of the FXR1 protein (1-379 aa) is highly conserved in mammals. The amino acid sequence of the FXR1 protein of Wistar rats used in this work was identified and annotated in GenBank (accession number MG938503). This sequence was compared with corresponding canonical sequences of the FXR1 protein of different vertebrate species presented in UniProt database (http://www.uniprot.org). Sequence alignment for the FXR1 N-terminal domains of ten vertebrate species are presented. Residues matching those in rat sequence are shaded in grey, whereas amino acid substitutions are shaded in red.

Supplementary Table S1. Primers used in this work

Primer	Sequence
fxr1 EcoRI forward	atagaattcggcggagctgacggtgga
fxr1(379) BamHI reverse	ataggatccttacataccaatctgtcgc
fxr1 HindIII forward	tacaagcttatggcggagctgacggt
fxr1 (YFP)BamHI reverse	tatggatcccataccaatctgtcgcagctg
fxr1 (380) HindIII forward	attaagcttatgggtttcagaccttct
fxr1 (568) BamHI reverse	tacggatccatcacatcttttgcctagc
fxr1 NotI forward	atagcggccgcagagctgacggtggaggt
fxr1 XbaI reverse	gtattctagattaaccaatctgtcgcagct
